# Interventions to Improve Communication at Hospital Discharge and Rates of Readmission: A Systematic Review and Meta-analysis

**DOI:** 10.1001/jamanetworkopen.2021.19346

**Published:** 2021-08-27

**Authors:** Christoph Becker, Samuel Zumbrunn, Katharina Beck, Alessia Vincent, Nina Loretz, Jonas Müller, Simon A. Amacher, Rainer Schaefert, Sabina Hunziker

**Affiliations:** 1 Medical Communication, Department of Psychosomatic Medicine, University Hospital Basel, Basel, Switzerland; 2 Emergency Department, University Hospital Basel, Basel, Switzerland

## Abstract

**Importance:**

Shortcomings in the education of patients at hospital discharge are associated with higher risks for treatment failure and hospital readmission. Whether improving communication at discharge through specific interventions has an association with patient-relevant outcomes remains unclear.

**Objective:**

To conduct a systematic review and meta-analysis on the association of communication interventions at hospital discharge with readmission rates and other patient-relevant outcomes.

**Data Sources:**

PubMed, EMBASE, PsycINFO, and CINAHL were systematically searched from the inception of each database to February 28, 2021.

**Study Selection:**

Randomized clinical trials that randomized patients to receiving a discharge communication intervention or a control group were included.

**Data Extraction and Synthesis:**

Two independent reviewers extracted data on outcomes and trial and patient characteristics. Risk of bias was assessed using the Cochrane Risk of Bias Tool. Data were pooled using a random-effects model, and risk ratios (RRs) with corresponding 95% CIs are reported. This study followed the Preferred Reporting Items for Systematic Reviews and Meta-analyses (PRISMA) reporting guideline.

**Main Outcomes and Measures:**

The primary outcome was hospital readmission, and secondary outcomes included adherence to treatment regimen, patient satisfaction, mortality, and emergency department reattendance 30 days after hospital discharge.

**Results:**

We included 60 randomized clinical trials with a total of 16 070 patients for the qualitative synthesis and 19 trials with a total of 3953 patients for the quantitative synthesis of the primary outcome. Of these, 11 trials had low risk of bias, 6 trials had high risk of bias, and 2 trials had unclear risk of bias. Communication interventions at discharge were significantly associated with lower readmission rates (179 of 1959 patients [9.1%] in intervention groups vs 270 of 1994 patients [13.5%] in control groups; RR, 0.69; 95% CI, 0.56-0.84), higher adherence to treatment regimen (1729 of 2009 patients [86.1%] in intervention groups vs 1599 of 2024 patients [79.0%] in control groups; RR, 1.24; 95% CI, 1.13-1.37), and higher patient satisfaction (1187 of 1949 patients [60.9%] in intervention groups vs 991 of 2002 patients [49.5%] in control groups; RR, 1.41; 95% CI, 1.20-1.66).

**Conclusions and Relevance:**

These findings suggest that communication interventions at discharge are significantly associated with fewer hospital readmissions, higher treatment adherence, and higher patient satisfaction and thus are important to facilitate the transition of care.

## Key Points

**Question:**

Are communication interventions at hospital discharge associated with rates of hospital readmission?

**Findings:**

In this systematic review and meta-analysis including a pooled analysis of 19 randomized clinical trials involving 3953 patients for the primary end point, communication interventions at discharge were significantly associated with lower readmission rates, higher medication adherence, and higher patient satisfaction.

**Meaning:**

These findings suggest that communication interventions at discharge have the potential to decrease hospital readmissions and improve treatment adherence and patient satisfaction.

## Teaser

This systematic review and meta-analysis examines the associations of communication interventions at hospital discharge with readmission rates and other patient-relevant outcomes.

## Introduction

Hospital discharge is a multidisciplinary process during which patients receive complex medical information and follow-up instructions. At discharge, health care practitioners need to explain critical information, such as patients’ diagnoses and their treatment, while integrating patients’ conditions, perceptions, and needs at the same time. However, patients may not understand or remember the information provided, resulting in confusion, misinterpretation and mismanagement of treatment regimen.^1,2^ Low health literacy, anxiety, cognitive impairment, or language barriers might further limit patients’ ability to understand medical information shared at discharge, resulting in treatment failures.^3^ Previous studies found that a clinically relevant proportion of patients being discharged from hospitals are not able to recall their diagnoses and discharge instructions.^4,5^ Shortcomings in the education of patients before hospital discharge have been associated with higher risk for hospital readmission^6^ and mortality.^7,8^

Unplanned hospital readmissions may indicate poor quality of care. According to the US national health insurance program, Medicare, 15% of patients discharged from the hospital are readmitted within 30 days, and 1 in 4 of those readmissions is potentially preventable.^9,10^ Unplanned readmissions costs are estimated at $20 billion in the United States annually.^11^ This has led medical authorities to look for interventions to improve the transition of care and penalize hospitals for readmission.^12^ While several factors influence the risk of hospital readmissions, shortcomings in the education of patients at hospital discharge may be one of the main modifiable factors. Still, there is insufficient evidence that improving discharge communication results in lower readmission rates and other patient-relevant outcomes.

In this study, we performed a systematic review and meta-analysis of randomized clinical trials (RCTs) that examined the effect of communication interventions in medical patients at hospital discharge on patient-relevant outcomes. We were especially interested in the association of communication interventions with readmission to hospital, adherence to treatment regimens, and patient knowledge 30 days after discharge.

## Methods

### Types of Studies, Participants, and Outcome Measures

This systematic review and meta-analysis was registered with the International Prospective Register of Systematic Reviews (PROSPERO, CRD42020146415). We followed the Preferred Reporting Items for Systematic Reviews and Meta-analyses (PRISMA) reporting guideline.^13^

We included RCTs in which the effect of any communication intervention for readmission, medical adherence, mortality, satisfaction, medical knowledge, or reattendance to the emergency department (ED) was assessed. Studies were eligible if the communication intervention was performed shortly before or at hospital discharge, if they had a randomized clinical design, included medical patients, and the intervention was conducted at hospital discharge. Studies conducted in surgical wards, psychiatry hospitals, or outpatient settings were excluded. Studies with interventions that continued after hospital discharge (eg, continuous teaching sessions) were also excluded.

### Search Methods for Identification of Studies

We used a comprehensive search strategy consisting of a combination of subject headings and free words. We searched PubMed, as well as Embase, CINAHL, and PsycInfo via Ovid. To enhance quality, the electronic search strategy was reviewed according to Peer Review of Electronic Search Strategies^14^ by a librarian who specialized in systematic reviews. As we only focused on RCTs, we incorporated a sensitivity and precision-maximizing RCT filter in our search strategy.^15^ The final search strategy for PubMed, which was adapted according to each database’s syntax, is available in the eAppendix in the Supplement. To identify additional published, unpublished, and ongoing studies, we tracked relevant references through Web of Science’s and PubMed’s cited reference search, applied the similar articles search of PubMed, and screened all references for potentially eligible studies. The data search was performed between August 2020 and February 2021, last performed on February 28, 2021.

### Study Selection

Two review authors (C.B. and S.Z.) screened titles and abstracts, which were found through the systematic search strategy, independently. Studies were included or excluded according to the inclusion criteria. Later, C.B. and S.Z. reviewed the full texts of studies considered eligible for inclusion independently, and disagreement was resolved by discussion and consensus.

### Data Extraction and Assessment of Methodological Quality

Two authors (C.B. and S.Z.) independently extracted the data of the included studies. Two authors (C.B. and S.Z.) independently assessed the included RCTs for methodological quality using the Cochrane Risk of Bias Tool.^16^ In cases of disagreement, consensus was reached by discussion. If necessary, a third author (S.H.) was consulted.

### Primary and Secondary End Points

Readmission to the hospital was the primary end point of our meta-analysis because the aim of communication interventions at discharge was to prevent this adverse outcome. Secondary end points were defined as adherence to treatment regimen, satisfaction, mortality, and knowledge of medication or diagnoses assessed 30 days after hospital discharge.

### Statistical Analysis

We express dichotomous data risk ratios (RRs) with 95% CIs. Data were pooled using a random-effects model. Heterogeneity of studies was identified through visual inspection of the forest plots. We used the *I*^2^ statistic, which quantifies inconsistency across studies, to assess the consequences of heterogeneity on the meta-analysis. An *I*^2^ of 50% or greater indicates a high level of heterogeneity. If data were not suitable for direct comparison, we applied narrative synthesis. Also, we assessed for evidence of publication bias (small-study effects). This was assessed visually using the funnel plot and quantitatively using some statistical tests (eg, Egger test).

For the primary and secondary end points, we determined the associations within each predefined subgroup and compared the summary associations across subgroups using random-effect models.^17^ Results were stratified based on type of intervention (medication counseling vs education on patient’s disease and its management vs specific communication techniques [ie, shared decision-making, motivational interviewing, and teach-back]), patient handout (additional written material vs no written material), age (>65 years vs ≤65 years), the proportion of women in the trial (≤50% women vs >50% women), location of study (US vs Europe vs other), risk of bias according to the Cochrane Risk of Bias Tool (poor or fair vs good), study setting (hospitalized patients vs ED patients) and primary disease (cardiology vs respiratory vs other). Cutoffs for stratification were chosen post hoc based on the distribution among trials to achieve a balanced number of patients per group.

We performed statistical analyses in Stata MP version 15.1 (StataCorp) using the METAN package. Two-sided *P* < .05 was considered statistically significant.

## Results

### Studies Identified

A total 15 778 of records were identified through our database search, and 3 additional records^18,19,20^ were found through other sources, such as cited references and similar-article search. We removed 5879 duplicates, discarded 7801 studies after examining titles, and discarded an additional 1843 studies after screening abstracts. Of the remaining 258 full-text articles, 60 studies^18,19,20,21,22,23,24,25,26,27,28,29,30,31,32,33,34,35,36,37,38,39,40,41,42,43,44,45,46,47,48,49,50,51,52,53,54,55,56,57,58,59,60,61,62,63,64,65,66,67,68,69,70,71,72,73,74,75,76,77^ were eligible for inclusion, with 19 trials^18,24,29,38,39,40,41,43,47,51,53,54,55,56,59,61,64,75,77^ included in quantitative analysis (eFigure in the Supplement).

### Description of Studies

Characteristics of studies are shown in Table 1 (included for quantitative analysis regarding the primary end point) and eTable 1 in the Supplement (all studies). Publication dates of the included studies range from 1981 to 2021. The studies were from 18 different countries, with 26 studies (43%) from the United States,^18,19,21,22,23,24,25,26,27,28,29,30,31,32,33,34,35,36,37,38,39,40,41,42,43,44^ 8 studies (13%) from the United Kingdom^45,46,47,48,49,50,51,52^ and 5 studies (8%) from Spain,^53,54,55,56,57^ and the remaining 21 studies (35%) from other countries. Across all 60 studies, a total of 16 070 participants were included, with a study sample size ranging from 25 to 3386 participants.

**Table 1.** Summary of the Included Studies Regarding the Primary End Point, With Quality Assessed Using the Cochrane Risk of Bias Tool

Study	Study purpose	Country	Participants	Design	Intervention	Control	Detailed communication/intervention elements	Outcomes, measures, and results	Risk of bias^a^
**Intervention: medication counseling**									
Smith et al,^47^ 1997	To investigate how seamless pharmaceutical care could be delivered and how to maintain a patient's therapeutic management plan across the secondary and primary interface	UK	Elderly medical patients being discharged with high probability of difficulties with their medication plan (N = 66)	Single-center RCT	Oral counseling by a pharmacist on medication and written pharmaceutical care plan to be shown to the pharmacist or physician (n = 34)	Usual care (summary of medication plan and written instructions for the GP**)** (n = 32)	Intervention: oral counseling by a study pharmacist on reason for medication, time of medication intake, side effects, importance of adherence, and how to arrange a new supply Control: written pharmaceutical care plan to be shown to the GP or community pharmacist Telephone helpline if help or advice during the first 7 d is needed	Primary: Adherence (10 d): 10 patients (40%) in the control group vs 23 patients (82%) in the intervention group showed adherence Secondary: Readmission (10 d): 1 patient (3%) in the control group vs 2 patients (6%) in the intervention group Death (10 d): 4 patients (13%) in the control group vs 1 patient (3%) in the intervention group	High
Sáez De La Fuente^55^ 2011	To evaluate the utility of a post-discharge pharmaceutical care program	Spain	Medical inpatients who are polymedicated with existing treatment for ≥3 mo prior to hospitalization and ≥4 active medications at discharge (N = 59)	Single-center RCT	Verbal and written pharmacotherapeutic information (n = 29)	Usual care (n = 30)	Verbal and written information about their treatment at hospital discharge	Primary: Adherence (30 d, Morisky Green-test): 15/24 patients (63%) in the control group vs 23/26 patients (88%) in the intervention group were adherent Secondary: Death (30 d): 1 patient (3%) in the control group vs 2 patients (7%) in the intervention group ED reattendance (30 d): 9 patients (30%) in the control group vs 7 patients (24%) in the intervention group Readmission (30 d): 7 patients (23%) in the control group vs 5 patients (17%) in the intervention group Modifications to treatment (30 d): no significant difference between the groups	Unlcear
Press et al,^24^ 2012	Effect of teach-back on the correct use of respiratory inhalers	US	Patients hospitalized with asthma or COPD (N = 50)	Single-center RCT	Oral and written information regarding inhalers + teach-to-goal (n = 24)	Oral and written information only (n = 26)	Patients in intervention group received demonstration of correct use of inhaler, further evaluation of patients' technique, written information	Primary: knowledge (metered dose inhaler) (30 d): 12 patients (46%) in the control group vs 3 patients (13%) in the intervention group (*P *= .01) Secondary: Readmission (30 d): 5/20 patients (25%) in the control group vs 1/19 patients (5%) in the intervention group Death (30 d): 3/20 patients (15%) in the control group vs 0/19 patients in the intervention group	Low
Sanchez Ulayar^53^ 2012	To determine the effectiveness of a pharmaceutical intervention with the patient on hospital discharge and to improve understanding of pharmaceutical treatment and adherence to medication at home	Spain	Medical inpatients who were polymedicated (N = 100)	Single-center RCT	Pharmacist counseling and personalized medication plan (n = 50)	Usual care (n = 50)	A pharmacist explained the medication prescribed, giving the patient a personalized medication timetable (with prescribed medication and when and which dose to take).The pharmacist explained why each medicine had been prescribed, how to take it, and why it was important to take the medication correctly.	Primary: adherence (7 d): 8/41 patients (20%) in the control group vs 29/41 patients (71%) in the intervention group (*P *< .001) Secondary: Death (30 d): no significant difference Readmission (30 d): 10/41 patients (24%) in the control group vs 3/41 patients (7%) in the intervention group (*P *< .05) Readmission (60 d): 13/41 patients (32%) in the control group vs 3/41 patients (7%) in the intervention group (*P *< .01)	High
Marušić et al,^59^ 2013	To evaluate the impact of pharmacotherapeutic counseling on the rates and causes of 30-d postdischarge hospital readmissions and ED visits	Croatia	Elderly medical patients (≥65 y) prescribed with ≥2 medications for chronic diseases (N = 160)	Single-center RCT	Predischarge counseling by the clinical pharmacologist about each prescribed medication (n = 80)	Usual care (n = 80)	Information about each prescribed medication was given: indications for prescription, dosage and time of intake, importance of adherence, possible consequences of nonadherence, ADE, prevention of ADEs, and measures to be taken in case of ADEs	Primary: readmission (30 d): 5 patients (6%) in the control group vs 6 patients (8%) in the intervention group (*P* = .75) Secondary: ED reattendance (30 d): no significant difference between groups Adherence (30 d): 43 patients (54%) in the control group vs 71 patients (89%) in the intervention group (*P* < .001) ADEs (30 d): no significant difference between groups (*P* = .32) Death (30 d): 2 patients (3%) in the control group vs 0 patients in the intervention group	Low
Press et al,^29^ 2016	Effects of 2 different educational strategies (teach-to-goal instruction vs brief verbal instruction) in adults hospitalized with asthma or COPD	US	Inpatients with asthma or COPD (N = 120)	Multicenter RCT	Oral and written information regarding inhalers + teach-to-goal (n = 62)	Oral and written information only (n = 58)	Patients in the intervention group received demonstration of correct use of inhaler, further evaluation of inhaler technique, and written information	Primary: knowledge (prevalence of inhaler misuse) (30 d): no significant difference between groups Secondary: Knowledge (prevalence of inhaler misuse) (90 d): misuse in intervention group was significantly lower than control group (25/ 52 patients [48%] vs 39/51 patients [76%]; *P* = .004) ED reattendance: 9/54 patients (17%) in the intervention group vs 16/53 patients (30%) in the control group Readmission (30 d): 6/54 patients (11%) in the intervention vs 13/53 patients (25%) in the control group	Low
Sanii et al,^75^ 2016	Effect of patient counseling at discharge on treatment satisfaction and medication adherence	Iran	Inpatients in the respiratory ward (N = 200)	Single-center RCT	Pharmacist counseling and education about prescribed medications (n = 78)	Usual care (n = 76)	Patients were educated on and informed about health conditions and drug therapy (medication counseling on all prescribed medications), side effects, inhaler technique assessment, and education Comparison of discharge medication with preadmission regimens Screening of previous drug-related problems (nonadherence, ADEs) Review of indications, directions for use, interactions, importance of adherence to medication, potential ADEs	Primary: adherence rate (30 d): mean (SD), 50.3% (27.1%) in the control group vs 93.2% (9.2%) points in the intervention group (*P* = .01) Secondary: Satisfaction (30 d): mean (SD) satisfaction score: 50.0 (16.2) points in the control group vs 83.5 (13.7) points in the intervention group (*P* = .01) Readmission (30 d): 8 patients (11%) in the control group vs 0 patients in the intervention group	High
Al-Hashar et al,^64^ 2018	Impact of medication reconciliation and counselling intervention on ADEs after discharge	Oman	Medical inpatients (N = 587)	Single-center RCT	Medication reconciliation intervention (n = 286)	Usual care (n = 301)	Involvement of pharmacist to detect discrepancies and resolve them, provide bedside counseling regarding medication, and provide medication list with educational material	Primary: No. of preventable ADEs (30 d): 59 ADEs in the control group vs 27 ADEs in intervention group (*P* = .008) Secondary: Readmission (30 d): 44 patients (15%) in the control group vs 39 patients (10%) in the intervention group (*P* = .91) ED reattendance (30 d): no significant difference between groups Death (30 d): no significant difference between groups	Low
Marušić et al,^77^ 2018	To evaluate the impact of pharmacotherapeutic education on 30-d post-discharge medication adherence and adverse outcomes in patients with type 2 diabetes	Croatia	Patients with type 2 diabetes (N = 130)	Single-center RCT	Individual predischarge pharmacotherapeutic education (n = 65)	Usual care (received standardized diabetes education) (n = 65)	Intervention group received additional individual predischarge pharmacotherapeutic education about the discharge prescriptions in 30-min sessions conducted by a physician; patients received information regarding indications for medication, dosage, administration time, the importance of medication adherence, possible consequences of nonadherence, possible ADEs, prevention and early detection of ADEs, and measures to be taken if an ADR is suspected. All patients were given a leaflet containing the same information in writing.	Primary: adherence to medication (30 d): 41/61 patients (67%) in the control group vs 57/64 patients (89%) in the intervention group (*P* = .003) Secondary: Adverse outcome: no significant difference between groups Readmission (30 d): 8/61 patients (13%) in the control group vs 5/64 patients (8%) in the intervention group (*P* = .33) ED reattendance (30 d): no significant difference between groups Death (30 d): no significant difference between groups ADEs (30d): 25/61 patients (41%) in the control group vs 23/64 patients (36%) in the intervention group	High
Graabaek et al,^61^ 2019	Effect of a pharmacist-led medicines management model among older patients on medication-related readmissions	Denmark	Medical inpatients aged >65 y (N = 600)	Single-center RCT with 3-group parallel design	Basic intervention (n = 200) Extended intervention (n = 200)	Standard discharge procedure (n = 200)	Basic intervention: pharmacist-led medication review, including patient interview and medication reconciliation Extended intervention: basic intervention + patient counselling and a medication report at discharge	Primary: Medication-related readmission (30 d): 11 patients (6%) in the control group, 9 patients (5%) in the basic intervention group, and 5 patients (3%) in the extended intervention group Secondary: Death (30 d): no significant difference among groups Overall mortality (180 d): no significant difference among groups Overall readmission rate (30 d): no significant difference between the control group (67/198 patients [19%] in the control group vs 46/194 patients in the extended intervention group [24%]) (*P* = .09) Overall readmission rate (180 d): no significant difference among groups ED reattendance (180 d): no significant difference among groups	Low
**Interventions: education regarding disease and its management**									
Osman et al,^51^ 2002	To determine if a brief self-management program given during hospital admission reduces readmission	UK	Patients with acute asthma (N = 280)	Single-center RCT	Self-management program with an educational session and a written self-management plan (n = 135)	Usual care (n = 145)	Structured and educational self-management program by a trained respiratory nurse on 2 occasions during hospital stay regarding knowledge about asthma, methods to recognize and avoid risk factors, and basic information about medication, booklet with information, and written self-management plan (symptom and peak flow based) based on discharge medication for the immediate time after discharge	Primary: readmission (1 y): 38/140 patients (27%) in the control group vs 22 or 131 patients (17%) in the intervention group (*P* = .04) Secondary: Readmission (30 d): no significant difference between groups (4 patients [<1%] in the control group vs 1 patient [<1%] in the intervention group; *P* = .40) Morbidity (30 d): patients in the intervention group were more likely than control group patients to report no daytime wheeze, no night disturbance, and no activity limitation Satisfaction with explanation (30 d): 89/118 patients (76%) in the control group vs 108/108 patients (100%) in the intervention group (*P* < .001)	Low
Adamuz et al,^54^ 2015	Effect of an educational program for inpatients on health care utilization after discharge	Spain	Medical inpatients with CAP (N = 207)	Multicenter RCT	Education at discharge regarding CAP (n = 102)	Usual care (n = 105)	Educational program including two 30-min sessions conducted by nurses between 24-72 h before discharge regarding fluid intake, medication adherence, vaccination, and knowledge and management of disease; patients also received handout about self-management of CAP	Primary: Health care utilization (30 d): significantly reduced in intervention group ED reattendance (30 d): 27 patients (26%) in the control group vs 11 patients (11%) in the intervention group (*P* = .007) Hospital readmission (30 d): 18 patients (17%) in the control group vs 5 patients (5%) in the intervention group (*P* = .007) Secondary: Satisfaction (30 d): 19 patients (18%) in the control group vs 84 patients (82%) in the intervention group (*P* < .001) Knowledge about diagnosis (30 d): 21 patients (20%) in the control group vs 100 patients (98%) in the intervention group (*P* < .001) Adherence (30 d): no significant difference between groups	Low
Fuenzalida et al,^56^ 2015	To assess if a nurse-led education for patients with AFdischarged from the ED improved the patient understanding of arrythmia and its treatment and reduces the number of complications and arrythmia-related admissions	Spain	ED patients with AF (N = 240)	Single-center RCT	Nurse-led education and information leaflet about AF, its treatment, precautions to take, warning signs, and pulse-taking (n = 116)	Usual care (n = 124)	Nurse-led patient education about the basic aspects of arrhythmia, possible complications, its treatment, precautions to take, and alarming symptoms Instructions on how to take pulse manually and to do so at least once/wk Advice to visit their GP Personalized leaflet with information about the prescribed medication and a summary of the previously described information	Primary: combined (death and complications): 30 patients (24%) in the control group vs 16 patients (14%) in the intervention group (*P* = .04) Secondary: Knowledge about diagnosis (30 d): no significant difference between groups Death (90 d): 9 patients (7%) in the control group vs 6 patients (5%) in the intervention group Readmission (30 d): 15 patients (12%) in the control group vs 8 patients (7%) in the intervention group Readmission (90 d): 26 patients (21%) in the control group vs 13 patients (14%) in the intervention group (*P *= .04)	Unclear
Athar et al,^38^ 2018	Effect of image of IVC as personalized education approach on medication adherence	US	Inpatients with decompensated HF (N = 97)	Single-center RCT	Education and image of IVC (n = 50)	Usual care (only generic information) (n = 47)	Intervention group patients were shown their IVC images by the ultrasonographer, who also provided them with real-time scripted educational information. Information was tailored to the amount of distension of IVC. Patients in intervention group also received a laminated patient education tool	Primary: adherence to HF regimen (30 d): no significant difference between groups (mean [SD] score, 11.7 [3.0] vs 11.8 [2.8]; *P *= .90) Secondary: Readmission (30 d): no difference between groups (7/44 patients [16%] vs 7/46 patients [15%]; *P* = .93) ED reattendance (30 d): no significant difference between groups	Low
Breathett et al,^39^ 2018	Effect of tablet application for education on readmission rates	US	Inpatients with HF (N = 126)	Single-center RCT	Education by nurse practitioner enhanced by tablet application (n = 60)	Standard discharge with nurse practitioner (n = 66)	Education included one-on-one discussion of heart failure materials. Tablet application was an interactive audio-visual program that provided individualized education and flagged patient questions to medical staff and included information on 4 specific topics: HF overview, nutrition plan, importance of medication adherence, and lifestyle modification	Primary: readmission (30 d): no significant difference between groups (16/60 patients [27%] in the control group vs 7/53 patients (13%) in the intervention group; *P *= .08) Secondary: Satisfaction (30 d): no significant difference between groups Self-perceived knowledge of purpose of medication (30 d): no significant difference between groups Self-perceived knowledge regarding meaning of HF (30 d): no significant difference between groups	High
Jasinski et al,^40^ 2018	Effect of education of patients and family members on readmission rate	US	Inpatients with end-stage kidney failure (N = 120)	Single-center RCT	Family consultation (n = 60)	Usual care (n = 60)	Family consultation occurred at patient's bedside and included the physician reviewing patient and family understanding of events that caused the hospital admission, assessing cognitive impairment, discussing ways for the support person to assist the patient with medication adherence, and providing tailored information about health and risk factors	Primary: readmission (30 d): no significant difference between groups (19 patients [32%] in the control group vs 12 patients [20%] in the intervention group; *P* = .15) Secondary: Readmission (180 d): no significant difference between groups ED reattendance (30 d): 12 patients (20%) in the control group vs 8 patients (13%) in the intervention group	Low
**Interventions: specific communication techniques **									
Hess et al,^41^ 2012	To test the effect of a decision aid on patient knowledge, patient engagement in decision making, and proportion of patients admitted to hospital	US	ED patients with nontraumatic chest pain (N = 208)	Single-center RCT	Decision aid (pictograph with the pretest probability of an acute coronary syndrome) and shared decision-making (n = 101)	Usual care (n = 103)	Intervention patients reviewed a decision aid that described the rationale and results of the initial evaluation (echocardiogram, troponin), provided the rationale for further cardiac stress testing, and depicted on a pictograph the patient's pretest probability for acute coronary syndrome within 45 d and indicating management options. Participating clinicians were oriented on how to use the decision aid prior to study.	Primary: Knowledge about diagnosis (immediately after discharge): 3.0 (95% CI, 2.7-3.2) correct answers (out of 7) in the control group vs 3.6 (95% CI, 3.4-3.9) correct answers in the intervention group Knowledge about prognosis (immediately after discharge): 1 patient (1%) in the control group vs 24 patients (25%) in the intervention group correctly assessed 45-d risk of ACS (*P* < .001) Secondary: Decisional Conflict Scale (30 d): 43.3 (95% CI, 32.2-39.6) points in the control group vs 22.3 (95% CI, 18.1-26.4) points Trust in physician (30 d): 79.3 (95% CI, 75.4-83.2) points in the control group vs 83.4 (95% CI, 79.4-87.3) points in the intervention group Patient involvement (OPTION-Scale): 7.0 (95% CI, 5.9-8.1) points in the control group vs 26.6 (95% CI, 24.9-28.2) points in the intervention group Satisfaction (30 d): 41 patients (40%) in the control group vs 62 patients (61%) in the intervention group ED reattendance (30 d): no significant difference between groups Readmission (30 d): 0 patients in the control group vs 2 patients (2%) in the intervention group (*P* = .24) Admission to cardiac observation unit: 77/100 patients (77%) in the control group vs 58/100 patients (58%) in the intervention group (*P* < .001) Adverse events (30 d): no significant difference between groups Death (30 d): no significant difference between groups	Low
Hess et al,^18^ 2016	To test the effectiveness of the decision aid to improve patient knowledge and decrease unnecessary resource use	US	ED patients with chest pain (N = 898)	Multicenter RCT	Shared decision-making (n = 451)	Usual care (n = 447)	Use of a Cates plot as a decision aid depicting risk of having a heart attack within the next 45 d	Primary: Knowledge about diagnosis (immediately after visit) 3.6 (1.5) correct answers out of 8 questions in control group vs 4.2 (1.5) correct answers in the intervention group Knowledge about prognosis (immediately after discharge): 2 patients (0.4%) in the control group vs 10 patients (2.2%) in the intervention group correctly assessed their 45-d risk of ACS (*P *= .04) Secondary: Decisional conflict scale (30 d): mean (SD) score, 46.4 (14.8) points in the control vs 43.5 (15.3) points in the intervention, Trust in physician (30 d): mean (SD) score, 87.7 (16.0) in the control vs 89.5 (13.4) in the intervention Patient involvement (OPTION-Scale): mean (SD) score, 7.9 (5.4) points in the control group vs 18.3 (9.4) points in the intervention group Readmission (30 d): 19 patients (5%) in the control group vs 20 patients (4%) in the intervention group (*P *= .88) ED reattendance (30 d): no significant difference between groups Adverse events (30 d): no significant difference between groups Death (30 d): no significant difference between groups Patient satisfaction (30 d): 192 patients (43%) in the control group vs 221 patients (49%) in the intervention group	Low
Eyler et al,^43^ 2016	Effects of motivational interview on medication adherence performed by pharmacists	US	Medical inpatients with pneumonia (N = 30)	Single-center RCT	Motivational interviewing-enhanced discharge care (n = 16)	Standard discharge procedure (n = 14)	Motivational interviewing and counseling on their antibiotics by a pharmacist and an assessment of readiness of discharge and confidence in adherence	Primary: adherence (7 d): no significant difference between groups (9 patients [64%] in the control group vs 14 patients [88%] in the intervention group; *P* = .14) Secondary: General satisfaction (30 d): patients were very satisfied with intervention Readmission (30 d): no difference between groups (4 patients [29%] in the control group vs 4 patients [25%] in the intervention group; *P* = .83)	High

Abbreviations: ACS, acute coronary syndrome; ADE, adverse drug event; AF, atrial fibrillation; CAP, community-acquired pneumonia; COPD, chronic obstructive pulmonary disorder; ED, emergency department; GP, general practitioner; HF, heart failure; IVC, inferior vena cava; OPTION, Observing Patient Involvement; RCT, randomized controlled trial; UK, United Kingdom.

^a^ Quality was assessed using the Cochrane Risk of Bias Tool.

A total of 24 studies (35%) recruited medical inpatients,^19,21,22,27,30,40,46,47,48,49,52,53,55,58,59,60,61,62,63,64,65,66,67,77^ of which 5 studies (24%) focused on patients aged 65 years or older^21,47,59,60,61^ and 5 studies (24%) focused on polymedicated patients,^22,46,53,55,58^ whereas 13 studies (22%) recruited ED patients.^18,25,28,31,32,34,35,41,42,44,56,57,68^ Thirteen studies (22%)^23,33,36,37,38,39,45,69,70,71,72,73,74^ recruited cardiology patients and 10 studies (17%)^20,24,29,43,50,51,52,54,75,76^ focused on patients with respiratory diseases.

In 28 studies (47%),^19,21,22,23,24,25,26,27,28,29,30,45,46,47,48,53,55,58,59,60,61,62,63,64,67,69,75,77^ the intervention focused on medication counseling, which was often conducted with the involvement of a pharmacist and consisted of education regarding dosage, the importance of medication adherence, and possible side effects. In 27 studies (45%),^31,32,33,34,35,36,37,38,39,40,44,49,50,51,52,54,56,57,65,66,68,70,71,72,73,74,76^ patients in the intervention groups were educated regarding their disease and its management. Study personnel focused on patient knowledge regarding their disease, prognosis, and complications, as well as self-management, such as fluid intake or lifestyle modification. There were 5 studies (8%)^18,20,41,42,43^ that used specific communication strategies, such as motivational interviewing, teach-back, or shared decision-making, as their interventions to encourage treatment adherence or patient involvement in decision-making. In total, we determined 19 studies (32%) were at low risk of bias, 32 studies (53%) were at high risk of bias, and 9 studies (15%) were at unclear risk of bias (eTable 2 in the Supplement).

### Quantitative Analysis

#### Readmission to Hospital Within 30 Days After Discharge

**Figure 1.**
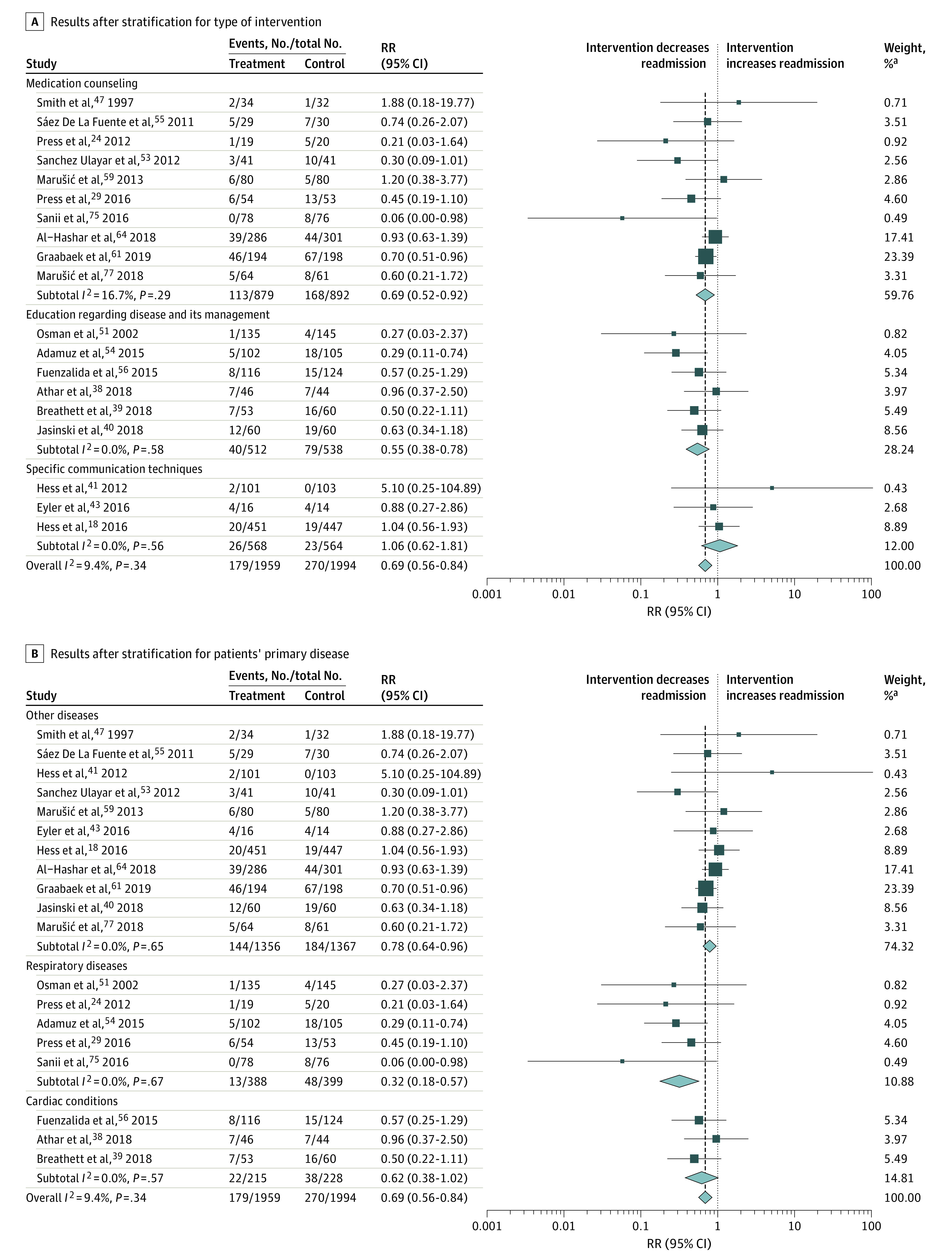
Forest Plots for the Associations of Communication Interventions With Readmissions Boxes indicate rate ratios (RRs); whiskers, 95% CIs; diamonds, pooled RR of readmission; vertical dashed lines, overall pooled RR of 0.69. ^a^Weights are from random-effects analysis.

Of 60 studies, 19 studies (32%)^18,24,29,38,39,40,41,43,47,51,53,54,55,56,59,61,64,75,77^ reported data regarding readmission within 30 days after hospital discharge and were thus included in the quantitative analysis. Regarding bias, 11 trials (58%) had low risk of bias, 6 trials (32%) had high risk, and 2 trials (11%) had unclear risk. There was no evidence for publication bias (Egger test: *P* = .21). Compared with usual care, the pooled results showed a significant association between communication interventions and fewer readmissions to hospital (179 of 1959 patients [9.1%] in intervention groups vs 270 of 1994 patients [13.5%] in control groups; RR, 0.69; 95% CI, 0.56-0.84). There was low heterogeneity among trials (*I*^2^ = 9.4%; *P* = .34) (Figure 1).

With regard to the primary end point, we performed a subgroup analysis stratified for the type of intervention, age, primary disease, use of handouts, study quality, setting of the study, sex, and location of study (Table 2). There was a significant subgroup difference regarding the primary disease of patients with trials including patients with respiratory conditions (RR, 0.32; 95% CI, 0.18-0.57) and patients with other illnesses (RR, 0.78; 95% CI, 0.64-0.96), whereas trials including patients with cardiac conditions showed no significant difference (RR, 0.62; 95% CI, 0.38-1.02; between-group heterogeneity: *P* = .01). We also found that trials with less than 50% of patients being women, compared to studies with more than 50% women patients, had better outcomes in hospital admission (RR, 0.55; 95% CI, 0.39-0.77 vs 0.82; 95% CI, 0.64-1.06; between-group heterogeneity: *P* = .08). Stratification by other subgroups did not show any differences in associations.

**Table 2.** Results After Stratification of Meta-analysis Regarding the Primary and Secondary End Points

Subgroup	Readmission				Adherence to treatment regimen					Satisfaction					Mortality					ED reattendance				
	Trials, No.	RR (95% CI)	Test for heterogeneity		Trials, No.	RR (95% CI)	Test for heterogeneity		Trials, No.		RR (95% CI)	Test for heterogeneity		Trials, No.		RR (95% CI)	Test for heterogeneity		Trials, No.		RR (95% CI)	Test for heterogeneity	
			*I*^2^, %	*P* value			*I*^2^, %	*P* value				I^2^, %	*P* value				I^2^, %	*P* value				I^2^, %	*P* value
Overall	19	0.69 (0.56-0.84)	9.4	.34	15	1.24 (1.13-1.37)	85.3	<.001	11		1.41 (1.20-1.66)	91.1	<.001	11		0.70 (0.38-1.29)	0.0	.79	11		0.86 (0.67-1.10)	48.3	.04
Stratified by type of intervention																							
Medication counseling	10	0.69 (0.52-0.92)	16.7	.29	NA	NA	NA	NA	2		2.30 (0.45-11.85)	95.2	<.001	NA		NA	NA	NA	6		0.87 (0.67-1.13)	36.5	.16
Education regarding disease and its management	6	0.55 (0.38-0.78)	0.0	.58	NA	NA	NA	NA	6		1.63 (1.14-2.32)	96.3	<.001	NA		NA	NA	NA	3		0.62 (0.36-1.07)	30.1	.24
Specific communication strategies	3	1.06 (0.62-1.81)	0.0	.56	NA	NA	NA	NA	3		1.16 (0.91-1.47)	84.5	.002	NA		NA	NA	NA	2		1.60 (0.56-4.59)	19.8	.26
Between-group heterogeneity				.12				NA					.02										.03
Stratified by patient handout																							
Additional written material	13	0.68 (0.55-0.85)	4.5	.40	12	1.21 (1.10-1.34)	87.0	<.001	7		1.59 (1.25-2.03)	93.5	<.001	8		0.71 (0.35-1.44)	0.0	.54	8		0.80 (0.61-1.05)	47.7	.06
No written material	6	0.71 (0.44-1.16)	30.9	.20	3	1.49 (1.12-1.97)	0.0	.74	4		1.25 (0.99-1.57)	86.5	<.001	3		0.69 (0.21-2.24)	0.0	.87	3		1.13 (0.57-2.22)	44.9	.16
Between-group heterogeneity				.80				.01					.52					.97					.13
Stratified by age, y																							
>65	10	0.67 (0.53-0.85)	0.0	.58	8	1.58 (1.08-2.29)	95.5	<.001	NA		NA	NA	NA	7		0.59 (0.26-1.33)	0.0	.73	5		0.69 (0.50-0.94)	1.3	.40
≤65	9	0.67 (0.46-0.98)	33.0	.15	7	1.05 (0.97-1.14)	62.9	.01	NA		NA	NA	NA	4		0.88 (0.35-2.17)	0.0	.53	6		0.98 (0.72-1.34)	51.6	.07
Between-group heterogenity				.53				.03					NA					.53					.03
Stratified by sex																							
≤50% of participants women	7	0.55 (0.39-0.77)	24.2	.24	8	1.35 (1.05-1.73)	90.1	<.001	5		2.45 (0.92-6.53)	98.5	<.001	6		0.86 (0.35-2.12)	0.0	.88	3		0.57 (0.37-0.88)	0.0	.44
>50% of participants women	11	0.82 (0.64-1.06)	0.0	.59	3	1.30 (0.97-1.75)	90.9	<.001	5		1.18 (1.01-1.37)	80.9	<.001	4		0.62 (0.23-1.71)	10.4	.34	7		0.89 (0.67-1.18)	33.9	.17
Between-group heterogeneity				.08				.08					.12					.76					.06
Stratified by country																							
US	8	0.71 (0.52-0.97)	0.0	.44	6	1.03 (0.97-1.10)	47.7	.09	NA		NA	NA	NA	2		0.40 (0.05-2.97)	0.0	.35	NA		NA	NA	NA
Europe	9	0.64 (0.50-0.82)	0.0	.50	7	1.61 (1.07-2.43)	96.1	<.001	NA		NA	NA	NA	6		0.53 (0.20-1.43)	0.0	.62	NA		NA	NA	NA
Other	2	0.32 (0.02-5.17)	74.9	.046	2	1.61 (1.16-2.24)	0.0	.57	NA		NA	NA	NA	3		0.94 (0.41-2.16)	0.0	.68	NA		NA	NA	NA
Between-group heterogeneity				.38				.003					NA					.59					NA
Stratified by study quality																							
Poor (poor + fair)	8	0.56 (0.38-0.83)	0.0	.58	10	1.43 (1.16-1.76)	77.8	<.001	6		1.41 (1.07-1.86)	92.8	<.001	7		0.66 (0.29-1.50)	0.0	.77	2		0.86 (0.52-1.43)	0.0	.85
Good	11	0.73 (0.57-0.94)	20.9	.24	5	1.10 (1.00-1.21)	86.6	<.001	5		1.49 (1.16-1.93)	92.5	<.001	4		0.76 (0.31-1.85)	0.0	.41	9		0.85 (0.63-1.14)	58.3	.01
Between-group heterogeneity				.20				<.001					.01					.82					.73
Stratified by study setting																							
ED	3	0.88 (0.47-1.65)	25.3	.26	3	1.04 (0.97-1.11)	64.3	.06	5		1.20 (1.03-1.39)	66.6	.02	NA		NA	NA	NA	3		1.29 (1.02-1.64)	0.0	.51
Hospital	16	0.66 (0.54-0.82)	8.3	.36	12	1.42 (1.13-1.78)	91.7	<.001	6		1.72 (1.21-2.45)	97.0	<.001	NA		NA	NA	NA	8		0.72 (0.58-0.89)	0.0	.63
Between-group heterogeneity				.33				.04					.39					NA					<.001
Stratified by primary disease																							
Cardiac	3	0.62 (0.38-1.02)	0.0	.57	NA	NA	NA	NA	2		2.43 (0.19-31.55)	98.0	<.001	NA		NA	NA	NA	NA		NA	NA	NA
Respiratory	5	0.32 (0.18-0.57)	0.0	.67	NA	NA	NA	NA	3		1.78 (0.97-3.25)	98.5	<.001	NA		NA	NA	NA	NA		NA	NA	NA
Other	11	0.78 (0.64-0.96)	0.0	.65	NA	NA	NA	NA	6		1.23 (1.04-1.45)	81.8	<.001	NA		NA	NA	NA	NA		NA	NA	NA
Between-group heterogeneity				.01				NA					.16					NA					NA

Abbreviations: ED, emergency department; NA, not applicable; RR, risk ratio.

### Secondary End Points

#### Adherence to Treatment Regimen 30 Days After Discharge

**Figure 2.**
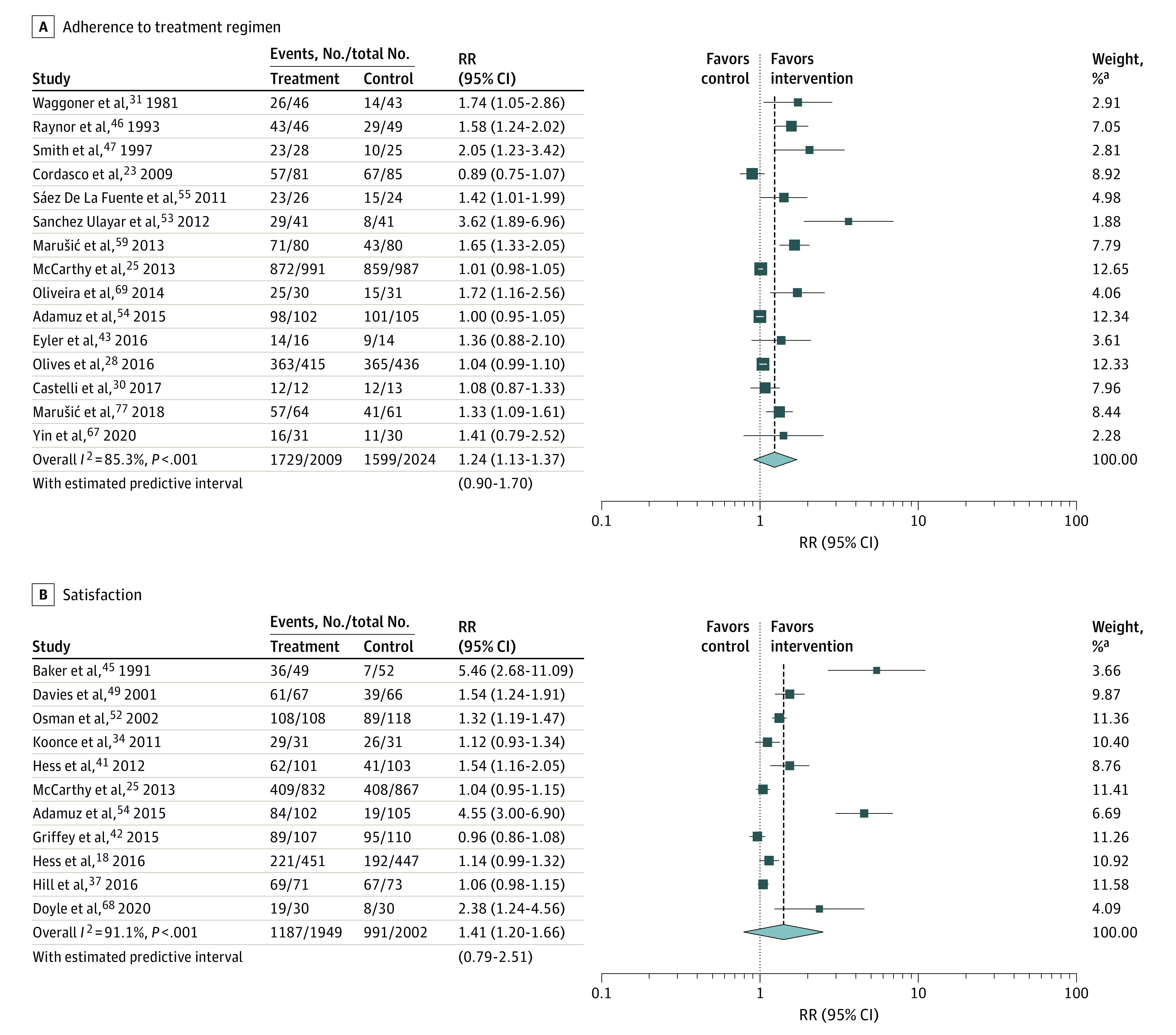
Forest Plots for the Associations of Communication Interventions With Secondary Outcomes Boxes indicate rate ratios (RRs); whiskers, 95% CIs; diamonds, pooled RR; vertical dashed lines, overall pooled RR of 1.24 (A) and 1.41 (B). ^a^Weights are from random-effects analysis.

Adherence to treatment regimen was assessed in 20 studies, with 15 studies (75%)^23,25,28,30,31,43,46,47,53,54,55,59,67,69,77^ reporting adherence in a dichotomous format (adherent vs not adherent), which we therefore pooled for a meta-analysis including 4033 patients. Regarding bias, 5 trials (33%) had a low risk of bias, 9 trials (60%) had a high risk, and 1 trial (7%) had an unclear risk. There was evidence for significant publication bias (Egger test: *P* = .006). The pooled analysis showed a significant association between a communication intervention at discharge and higher patient adherence to treatment 30 days after discharge (RR, 1.24; 95% CI, 1.13-1.37). There was substantial heterogeneity among trials (*I*^2^ = 85.3%; *P* < .001) (Figure 2A).

In a subgroup analysis, trials including older patients (ie, mean age >65 years) found associations of the intervention with adherence, whereas trials including younger patients (ie, ≤65 years) did not (RR, 1.58; 95% CI, 1.08-2.29 vs RR, 1.05; 95% CI, 0.97-1.14; between-group heterogeneity: *P* = .03). Also, in hospitalized patients, there was an association of the intervention with adherence, whereas in ED patients, there was no association (RR, 1.42; 95% CI, 1.13-1.78 vs 1.04; 95% CI, 0.97-1.11; between-group heterogeneity: *P* = .04). Of the 5 studies not included in the meta-analysis, 3 studies^26,55,75^ showed a significant increase in treatment adherence in patients who received a communication intervention.

#### Patient Satisfaction 30 Days After Discharge

A total of 15 studies (25%) evaluated the association of communication interventions with patients’ satisfaction within 30 days after hospital discharge. Of these, 11 studies (73%)^18,25,34,37,41,42,45,49,51,54,68^ reported satisfaction in a dichotomous format (satisfied vs not satisfied) and were thus pooled for a meta-analysis (including 3951 patients). Regarding bias, 5 trials (45%) had a low risk of bias, 5 trials (45%) had a high risk, and 1 trial (9%) had an unclear risk. There was evidence for significant publication bias (Egger test: *P* = .02).

Compared with usual care, the pooled analysis showed a significant association between communicational interventions and higher patient satisfaction (RR, 1.41; 95% CI, 1.20-1.66). There was substantial heterogeneity among trials (*I*^2^ = 91.1%; *P* < .001) (Figure 2B). A subgroup analysis found an association of the intervention with patient satisfaction in trials using medication counseling (RR, 2.30; 95% CI, 0.45-11.85; between-group heterogeneity: *P* = .02). Of the 4 studies not included in the meta-analysis, 1 study^75^ found a significant improvement of satisfaction in patients who received a communication intervention.

#### Mortality 30 Days After Discharge

We found 14 trials (23%) that assessed mortality of patients within 30 days after discharge. Of these, 3 studies^18,30,41^ did not report any deaths. The remaining 11 studies (23%),^20,24,26,47,53,55,59,61,64,76,77^ including 1787 patients. Of these, 4 trials (36%) had a low risk of bias, 5 trials (45%) had a high risk, and 2 trials (18%) had an unclear risk. In the quantitative analysis, there were no significant associations between communication interventions at discharge and mortality within 30 days (RR, 0.70; 95% CI, 0.38-1.29). There was no evidence for publication bias (Egger test: *P* = .10). There was no heterogeneity among trials (*I*^2^ = 0.0%; *P* = .79), and there were no differences in any subgroup analyses.

#### Reattendance to the ED

There were 11 studies (18%)^18,25,29,38,40,41,54,55,59,64,77^ that assessed ED reattendance that were included in the quantitative analysis, including 9 trials (27%) with a low risk of bias, 1 trial (9%) with a high risk, and 1 trial (9%) with an unclear risk. There was no evidence for publication bias (Egger test: *P* = .28). Overall, there was no significant association between communication interventions at discharge and ED reattendance (RR, 0.86; 95% CI, 0.67-1.10). There was a moderate heterogeneity among trials (*I*^2^ = 48.3%; *P* = .04).

#### Knowledge of Medication and Diagnoses 30 Days After Discharge

There were 22 trials (37%) that evaluated the effect of communication interventions on patient knowledge within 30 days after hospital discharge, including 6 trials (27%) with low risk of bias, 13 trials (22%) with high risk, and 3 trials (14%) with unclear risk. Of these. 11 studies (50%)^19,22,23,24,27,29,32,45,46,48,58^ evaluated patient knowledge of medication, 8 studies (36%)^18,34,35,41,54,56,72,73^ analyzed knowledge of diagnosis, and 3 studies (14%)^39,42,65^ evaluated both.

These studies used various interventions, such as medication counseling^19,22,23,24,27,29,45,46,58^ with reminder handouts, face-to-face counseling, or videos to educate about various aspects of the disease^32,34,35,39,54,56,65,72,73^ that were adapted, for example, to patients’ age, language, learning styles, or health literacy, or used specific communication strategies.^18,41,42^ Studies used either disease-specific knowledge questionnaires,^34,35,54,72,73^ and asked patients about their understanding of their diagnosis^39,56,65^ or their risk of adverse outcomes^18,41^ to assess patient knowledge. Studies that assessed knowledge of medication primarily counted medication errors,^24,29^ questioned patients about their treatment or its purpose,^19,23,39,45,46,58,65^ or used different structured questionnaires^22,27,32^ as their way of assessment. In summary, 13 studies (59%)^18,19,22,24,32,35,41,45,46,54,65,72,73^ found an association of communication interventions with an increase in knowledge in patients.

## Discussion

This systematic review and meta-analysis from 60 trials and 16 070 patients from 18 countries, including 19 RCTs and 3953 patients from 7 countries for analysis of the main outcome, found communication interventions at discharge to be associated with fewer hospital readmissions and improvement of treatment adherence and patient satisfaction. A subgroup analysis found associations in patients with respiratory illnesses regarding readmission, in older and hospitalized patients regarding adherence, and in trials using educational interventions regarding satisfaction.

This study confirms previous individual RCTs suggesting that communication interventions are highly effective in reducing hospital readmissions. Several reasons for preventable hospital readmissions have been proposed, including low adherence to following instructions or treatment regimens.^78,79^ We found that educational interventions, such as medication counseling or disease-specific education, were associated with lower readmission rates. This finding suggests that educating patients at discharge regarding their medication, diagnoses, or therapeutic regimen might partly explain the lower risk of readmissions. According to our results, patients with chronic conditions, such as respiratory illnesses, experienced the most benefit from communication interventions with regard to readmission rates. Patients with chronic conditions (eg, chronic obstructive pulmonary disease) rely on stringent treatment plans and are encouraged to actively participate in their care. Communication interventions with an educational or counseling approach might have highlighted the importance of adherence and thus explain the difference between the subgroups.

Previous research has highlighted the importance of family members in the discharge process of older patients, particularly those with frailty, delirium, or other cognitive deficits.^80^ Educational efforts in family members might also help to facilitate the discharge process in this cohort of patients with uniquely high risk. Compared with educational interventions, specific communication techniques, such as shared decision-making or motivational interviewing, were not associated with reduced readmission rates. In fact, 2 studies^18,41^ using shared decision-making in patients with low-risk chest pain reported an association with fewer hospitalization days. In consequence, this might have resulted in higher rates of readmission to hospital and reattendance to the ED.

Our data show that communication interventions were associated with an increase of adherence to treatment regimen. Adherence is known to be an independent factor associated with health-related outcomes, such as hospital readmission, mortality, morbidity, or quality of life.^81^ Especially in patients with chronic diseases, careful adherence to therapeutic regimens is of major importance, and approximately 40% of readmissions in these patients can be traced back to a lack of adherence.^81^ In a subgroup analysis, we found that communication interventions had were associated with better adherence in older and hospitalized patients from medical wards but not in younger patients and ED patients. This finding might be explained by the fact that most of these studies assessed interventions with an educational approach, as knowledge of disease and the purpose of the medication is known to be associated with adherence to therapeutic regimens in older adults.^82,83^

Although adherence is considered a requirement for a successful treatment and several of its barriers are potentially modifiable, the topic is commonly not addressed during physician-patient encounters.^84^ Furthermore, physicians may not be able to estimate whether their patient adheres to the prescribed treatment.^84^ Thus, communication strategies at discharge addressing patient knowledge might improve patient adherence.

Communication interventions at discharge were associated with improved patient satisfaction. Previous research has shown that patient satisfaction may not only affect patient outcomes but may also prevent complaints or even malpractice claims.^85^ Today, patient satisfaction is considered an important quality indicator in health care. Regulations in several countries, including the US, increasingly require that patients’ hospital experience be assessed through patient-related experience measurements, such as the Hospital Consumer Assessment of Healthcare Providers and Systems (HCAHPS).^86^ HCAHPS assesses patients’ perception of care delivered, and high patient satisfaction is linked to favorable HCAHPS metrics. Hospitals’ HCAHPS results are publicly accessible on the internet and not only influence a hospital’s reputation but also their reimbursements for care provided, which is why improving patient satisfaction has moved into the spotlight of health care systems. Our analysis suggests that communication interventions at discharge may improve quality of care and be considered a cost intervention. Still, cost-effectiveness studies are needed to understand costs associated with resource use and cost-savings resulting from the improvements in outcomes.

The qualitative results of our systematic review suggest that communication interventions at discharge may help to increase patient knowledge regarding disease, its therapy, and further therapeutic regimen. It is known that patient knowledge is an independent factor associated with adherence to treatment regimens, which is of major importance for a patient’s recovery.^87^ Furthermore, with regard to patient-centered care, knowledge empowers patients to understand complex medical information and instructions and act accordingly. Hence, communication interventions at discharge might increase patient knowledge, helping patients to participate in clinical decision-making more effectively.

Finally, several discharge pathways include patient handouts as supplementary information during the discharge process. Two systematic reviews^88,89^ investigated the associations of discharge interventions that were facilitated by written information or information technology, such as videos, with patient comprehension and satisfaction. Newnham and colleagues^89^ investigated which hospital discharge communication practices were preferred by patients and health care practitioners and were associated with improved patient and practitioner satisfaction and increased patient understanding of their medical condition.^89^ Based on review of 30 trials (3489 patients), Newnham et al^89^ concluded that well-designed information technology solutions may improve communication, coordination, and retention of information. Hoek and colleagues^88^ aimed to provide an overview of the different manners of providing discharge instructions in the ED and to assess their associations with comprehension.^88^ Based on 51 included articles, Hoek et al^88^ concluded that communicating discharge instructions verbally to patients may not be sufficient and adding video or written information is needed. Our analysis focused on associations of communication interventions at hospital discharge with patient-relevant outcomes. Interestingly, in our subgroup analysis, interventions that had included written patient handouts did not show better patient outcomes in readmission or adherence compared with interventions with oral information only. Clearly, further research is needed to investigate optimal ways to combined oral and written information at discharge.

### Limitations

This study has several limitations. First, as we were interested in interventions to improve patient-centered hospital-based care, we only included studies that focused on adult medical inpatients and excluded studies with outpatients, pediatric studies, and studies conducted in an outpatient setting. Also, we focused on communication interventions in isolation from each other and did not assess the complexity of a multidisciplinary discharge process. In clinical practice, interventions are often combined, and different members of the interprofessional team may deliver distinct discharge education. This approach might limit the generalizability of our results. Second, the included studies are very heterogeneous concerning the assessment of some end points, such as knowledge, which only allowed us to conduct a qualitative assessment and thus limits our ability to draw a systematic conclusion. Furthermore, we found evidence of publication bias for some secondary end points. Therefore, prospective validation is warranted. Also, some of the variables we selected for the subgroup analyses (eg, age, sex) may suffer from aggregation bias. Furthermore, we focused on studies in which communication interventions had finished at hospital discharge and disregarded studies with ongoing interventions, such as follow-up appointments or reminder messages or phone calls, which might also influence the patient-relevant outcomes that we assessed in our meta-analysis. Additionally, social determinants of health, such as race and ethnicity, educational level, and economic status, have an important role in the discharge process and might influence hospital readmission, treatment adherence, or medical knowledge. However, most of the studies we included in our meta-analysis only provided limited sociodemographic information, which made it impossible to stratify our results for these determinants.

## Conclusions

The findings of this systematic review and meta-analysis suggest that communication interventions at discharge are associated with reducing hospital readmissions and improving treatment adherence and patient satisfaction. Communication interventions at hospital discharge are important to facilitate the transition of care. Thus, health care systems should implement such communication strategies at discharge to facilitate the transition of care.

## Notes

Supplement. **eAppendix.** Search Strategy for PubMed **eTable 1.** Summary of the Included Studies, With Quality Assessed Using the Cochrane Risk of Bias Tool **eTable 2.** Risk Assessment by Cochrane Risk of Bias Tool **eFigure.** Flow of Studies Through the Review Process
