# Supplementary material for: Interventions to Improve Communication at Hospital Discharge and Rates of Readmission: A Systematic Review and Meta-analysis
Source: JAMA Netw Open. 2021 Aug 27;4(8):e2119346. doi: 10.1001/jamanetworkopen.2021.19346 (PMC8397933; doi:10.1001/jamanetworkopen.2021.19346)
Supplement: Supplement. — eAppendix. Search Strategy for PubMed eTable 1. Summary of the Included Studies, With Quality Assessed Using the Cochrane Risk of Bias Tool eTable 2. Risk Assessment by Cochrane Risk of Bias Tool eFigure. Flow of Studies Through the Review Process [file jamanetwopen-e2119346-s001.pdf]

Supplemental Online Content

Becker C, Zumbrunn S, Beck K, et al. Interventions to improve communication at hospital discharge and rates of readmission: a systematic review and meta-analysis. *JAMA Netw Open*. 2021;4(8):e2119346. doi:10.1001/jamanetworkopen.2021.19346

eAppendix. Search Strategy for PubMed
eTable 1. Summary of the Included Studies, With Quality Assessed Using the Cochrane Risk of Bias Tool
eTable 2. Risk Assessment by Cochrane Risk of Bias Tool
eFigure. Flow of Studies Through the Review Process

This supplemental material has been provided by the authors to give readers additional information about their work.

© 2021 Becker C et al. *JAMA Network Open.*

eAppendix. Search Strategy for PubMed

(discharg*[tiab] OR “patient discharge”[mh]) AND (communicat*[tiab] OR discuss*[tiab] OR dialogue[tiab] OR educat*[tiab] OR "Patient Education as Topic"[mh:noexp] OR "Health Communication"[mh] OR “Patient Education Handout"[mh] OR teach*[tiab] OR train*[tiab] OR inform*[tiab] OR “health literacy”[tiab] OR “health literacy”[mh] OR “Medical Illustration"[mh] OR illustrat*[tiab] OR pamphlet*[tiab] OR Pamphlet*[mh] OR brochure*[tiab] OR booklet*[tiab] OR handout*[tiab] OR leaflet*[tiab] OR Counseling[mh] OR counsel*[tiab] OR “Reminder Systems”[mh] OR remind*[tiab] OR telephone[tiab] OR phone[tiab] OR postcard*[tiab] OR SMS[tiab] OR Whatsapp[tiab] OR letter*[tiab] OR message*[tiab] OR “motivational interviewing”[tiab]) AND (readmission[tiab] OR “patient readmission”[mh] OR reattendance[tiab] OR knowledge[tiab] OR Knowledge[mh] OR “patient activation”[tiab] OR adherence[tiab] OR “Treatment Adherence and Compliance”[mh] OR satisfaction[tiab] OR “patient satisfaction”[mh] OR mortality[tiab] OR mortality[mh] OR HCAHPS[tiab] OR quality[tiab]) AND (randomized controlled trial[pt] OR controlled clinical trial[pt] OR randomized[tiab] OR placebo[tiab] OR clinical trials as topic[mesh:noexp] OR randomly[tiab] OR trial[ti]) NOT (animals[mh] NOT (humans[mh] AND animals[mh]))

© 2021 Becker C et al. *JAMA Network Open.*

eTable 1. Summary of the Included Studies, With Quality Assessed Using the Cochrane Risk of Bias Tool

	Title	Source	Year	Study Purpose	Country	Participants	Design	Methods / Interventions		Detailed communication / Intervention Elements	Outcomes, Measures and Results	Risk of bias
Interventions: Drug counseling												
Baker, D et al	Evaluation of drug information for cardiology patients	British journal of clinical pharmacology	1991	To establish if specially prepared drug information leaflets improve the understanding and recall of information relevant to the administration of drugs	UK	Cardiology patients n=125	Single center RCT	**Intervention group** (n = 49)<br>Individualised drug information leaflets on discharge	**Control group** (n = 52)<br>Usual care (verbal drug counseling)	Individualised patient information leaflets about the respective cardiovascular drugs in addition to verbal drug counseling	**1EP:**<br>- Knowledge (Drug, 2 weeks): 21 (40.4%) in the control group knew the purpose of treatment vs. 43 (87.8%) in the intervention group<br><br>**2EP:**<br>- Satisfaction: 7 (13.5%) in the control group vs. 36 (73%) in the intervention group felt sufficiently informed	poor
Raynor, DK et al	Effects of computer generated reminder charts on patients' compliance with drug regimens	BMJ	1993	To investigate if a reminder chart improved patients' compliance with their drug regimen after discharge from hospital	UK	Medical patients being discharged with regular intake of 2 to 6 drugs n=197	Multicenter RCT 4-group parallel design	**Group 1** (n=50)<br>Counseling from nurse and reminder chart about medicines<br>**Group 2** (n=50)<br>Structured counselling from pharmacist about medicines<br>**Group 3** (n=48)<br>Structured counselling from pharmacist and reminder chart about medicines	**Control group** (n=49)<br>Brief counseling from nurse	Individualised, computer-generated reminder charts about the timing of medicine intake in addition to verbal drug counseling from a nurse (group B) or a pharmacist (group D) compared to verbal counseling from a nurse (group A) or pharmacist (group C) alone	**1EP:**<br>- Adherence (10d): 29/49 patients in the control group vs. 43/46 patients in the intervention group (group 3) reached a compliance score of >85% (derived from counting patients' tablets)<br><br>**2EP:**<br>- Knowledge (Drug, 10d): 23/49 patients in the control group vs. 41/48 in the intervention group (group 3) answered all drug-related questions correctly	poor

© 2021 Becker C et al. *JAMA Network Open.*

Esposito, L et al	The effects of medication education on adherence to medication regimens in an elderly population	J Adv Nurs	1995	Effect of medication education on adherence to regimen	USA	Medical inpatients older than 65 years n=42	Single center RCT¶ 4-group parallel design	**Group 1** (n=8) Verbal instructions regarding medication at discharge G**roup 2** (n=10) Written instruction (medication schedule) regarding medication at discharges G**roup 3** (n=14) Written and verbal instructions verbal instructions regarding medication at discharge	**Control group** (n=11) Standard discharge	10-20min medication education<br><br>Medication schedule with a) the name of the drug, b) the color of the pill c), the number assigned, d) the dose, e) the time the dose should to be taken, f) when to take the medication (l e breakfast), g) side-effects and h) reason for medication	**1EP:**<br>**-** Adherence (14d): Mean adherence score of 2.69 in control group vs. 1.91 in the intervention group (group 3)<br>- Adherence (30d): Mean adherence score of 2.69 in control group vs.1.94 in the intervention group (group 3)<br>- Adherence (60d): Mean adherence score of 2.67 in control group vs. 1.94 in the intervention group (group 3)<br><br>**2EP:**<br>- Knowledge (Drugs) (Medication errors, 60d): 5/11 patients in control group vs. 1/14 in group 3 made medication errors	poor
Smith, L et al	An investigation of hospital generated pharmaceutical care when patients are discharged home from hospital	British journal of clinical pharmacology	1997	To investigate how seamless pharmaceutical care could be delivered and how to maintain a patient's therapeutic management plan across the secondary and primary interface	UK	Elderly medical patients being discharged with high probability of difficulties with their medication plan n=66	Single center RCT	**Intervention group** (n=28) Oral counseling by a pharmacist on medication and written pharmaceutical care plan to be shown to the pharmacist/doctor	**Control group** (n=25) Usual care (summary of medication plan and written instructions for the GP)	Oral counseling by a study pharmacist on reason for medication, time of drug intake, side effects, importance of compliance and how to arrange a new supply<br><br>Written pharmaceutical care plan to be shown to the GP/community pharmacist<br><br>Telephone helpline if help/advice during the first 7 days is needed	**1EP:**<br>- Adherence (10d): 10 patients in the control group vs. 23 in the intervention group showed compliance after 10 days<br><br>2EP:<br>- Readmission (10d): 1/32 patients in the control group vs. 2/34 patients in the intervention group were readmitted<br>- Death (10d): 4/32 patients in the control group vs. 1/34 patients in the intervention group died	poor
Strobach, D et al	Patient medication counseling-Patientenberatung zur Entlassungsmedikation	Med Klin	2000	To assess the effect of medication counseling on patient knowledge	Germany	Adult patients with more than 3 drugs and malcompliance n=37	Single center RCT	**Intervention group** (n=16) Medication counseling by pharmacist and written information regarding drugs	**Control group** (n = 21) usual care	Face-to face counseling regarding indication, dosage and side-effects of drugs<br>Written information regarding name, dosage and indication	**1EP:**<br>**-** Knowledge (Drugs, at discharge): 36% of patients in the control group (n=20) vs. 64% of patients in the intervention group (n=13) knew the indication for their treatment<br>- Knowledge (Drugs, 14d): 40.5% of patients in the control group (n=12) vs.90% of patients in the intervention group (n=7) knew the indication for their treatment	poor

© 2021 Becker C et al. *JAMA Network Open.*

Manning, DM et al	3D: a tool for medication discharge education	Quality & safety in health care	2007	To test 3D (tool, Durable Display at Discharge) versus MDW (Medication Discharge Worksheet) in patient satisfaction, knowledge, and self-reported medication errors	USA	Patients with more than three discharge medications n=337	Single center RCT	**Intervention group** (n=78)<br>Education with 3D tool (Durable Display at Discharge)	**Control group** (n=60)<br>Usual care (Medication Discharge Worksheet (MDW) is a paper medication list and schedule given to the patient as standard medication discharge education)	Patient education by a nurse before discharge<br><br>3D tool is an extended medication list and schedule generated semi-automatically from a database of 900 medications. It indicates the time of intake, purpose, cautions and comments, a reconciliation prompt and space for durable display (in order to glue a sample of each pill onto the list). The font is enlarged compared to usual care.	**1EP:**<br>Patient satisfaction (14 days, scale from 1 (low) to 5 (high): Patients in the control group had a score of 4.26 (0.8768) vs. 4.24 (0.6986) in the intervention group, p=0.5204<br><br>**2EP:**<br>- Knowledge (Drug, 14 days) (scale from 0 (low) to 3 (high): Patients in the control group had a score of 1.66 (0.6851) vs. 1.96 (0.7561) in the control group, p=0.0282<br>- Medications errors made since discharge (score 0-4): Score in the control group of 0.79 (0.4113) vs. 0.78 (0.4187) in the intervention group, p=0.8760	poor
Cordasco, KM et al	A low-literacy medication education tool for safety-net hospital patients	American journal of preventive medicine	2009	To evaluate a low-literacy medication education tool to improve medication adherence in cardiac patients in partnership with a safety-net provider	USA	Patients with congestive heart failure or coronary heart artery disease from all health literacy levels with at least three medications at discharge n=286	Single center RCT	**Intervention group** (n=100)<br>Low-literacy medication tool customized to patients' prescribed medication	**Control group** (n=110)<br>Usual care	Discharge medication education by a nurse with the help of a low-literacy medication tool<br><br>Low literacy-medication tool with pill pictures, simple instruction-specific icons, schedule of medication intake and customized to the patients' prescribed medication and printed on color paper.<br>After the nurse education, the patient was encouraged to take the tool home.	**1EP**:<br>- Adherence (14d): 78.3% (95% CI: 72.1% to 84.4%) in the control group vs. 70.5% (95% CI: 62.2 %to 78.7%) in the intervention group were self-reportedly adherent, p=0.13<br><br>**2EP:**<br>- Doses reported as missed at 4 weeks post discharge: a mean of 0.46 (95% CI: 0.16-0.76) doses in the control group vs. 1.1 (95% CI: 0.6-1.6) in the intervention group were reported missed, p=0.03<br>- Knowledge (Drug, 14d): 55/85 patients in the control group vs. 52/81 patients in the intervention group could spontaneously name their drugs**,** p=not significant<br>- Knowledge (Drug, 14d): 26/85 patients in the control group vs. 28/81 patients in the intervention group correctly knew the purpose of their treatment, p=not significant	poor

© 2021 Becker C et al. *JAMA Network Open.*

Bladh, L et al	Effects of a clinical pharmacist service on health-related quality of life and prescribing of drugs: a randomised controlled trial	BMJ quality & safety	2011	To evaluate the effects of a clinical pharmacist service on health-related quality of life (HRQL) and prescribing of drugs	Sweden	Medical inpatients n=400	Single center RCT	**Intervention group** (n=164)<br>Pharmacist-led medication reviews with feedback to the physicians, drug treatment discussion with patients at discharge and medication reports	**Control group** (n=181)<br>Usual care	Pharmacists performed continuous medication reviews with feedback to the physicians to identify inappropriate prescriptions, drug treatment discussion with patients at discharge, medication reports given to the patients at discharge and sent to their GP. Drug related problems were identified and were classified.	**1EP:**<br>- Quality of life (6 months, EQ VAS): Patients in the control group had a EQ VAS of 56.3 (16.6) vs. 59.1 (17.0) in the intervention group, p=0.38<br><br>**2 EP:**<br>- Inappropriate prescriptions per patients (PIP) were compared in both groups (admission vs. discharge), no significant difference was found<br>- Drug related problems: multiple drug related problems were found (e.g. adverse reactions, dosing problems) in the intervention group	poor
Sáez De La Fuente , J et al	Efficiency of the information given at discharge and adherence of polymedicate d patients	Farmacia Hospitalari a	2011	To evaluate the utility of a post-discharge pharmaceutical care program	Spain	Polymedicat ed medical inpatients with existing treatment for at least 3 months prior to hospitalisati on and 4 or more active medications at discharge n=59	Single center RCT	**Intervention group** (n=29)<br>Verbal and written pharmacotherapeutic information	**Control group** (n=30)<br>Usual care	Verbal and written information about their treatment at hospital discharge following the model of the Inofwin program	**1EP:**<br>-Adherence (30d, Morisky Green-test): 15/24 patients in the control group vs. 23/26 patients in the intervention group were adherent to treatment at follow-up (OR 4.6, 95% CI: 1.1-19.8), p=0.03)<br><br>**2EP:**<br>- Death (30d): 1/30 patient in the control group vs. 2/29 patients in the intervention group died (OR 2.2., 95% CI: 0.19-26.1), p=0.51<br>- A&E Reattendance (30d): 9/30 patients in the control group vs. 7/29 patients in the intervention group reattended the ED (OR 0.8, 95% CI: 0.2-2.6), p=0.74<br>- Readmission (30d): 7/30 patients in the control group vs. 5/29 patients in the intervention group were readmitted (OR 0.7, 95% CI: 0.2-2.7), p=0.66<br>- Modifications to treatment (30d): 70% of patients had some change to treatment, although no significant difference between the groups regarding the causes for change were found	fair

© 2021 Becker C et al. *JAMA Network Open.*

Press, VG et al	Teaching the use of respiratory inhalers to hospitalized patients with asthma or COPD: A randomized trial	Journal of General Internal Medicine	2012	Effect of teach-back on the correct use of respiratory inhalers	USA	Patients hospitalized with asthma or COPD n=50	Single center RCT	**Intervention group** (n=24)<br>Oral and written information regarding inhalers plus teach-to-goal	**Control group** (n = 26)<br>Oral and written information only	Patients in intervention group received demonstration of correct use of inhaler, further evaluation of patients' technique, written information	**1 EP:**<br>**-** Knowledge (Drugs: Prevalence of inhaler misuse) (30d): 46% in the control group (information group) vs. 13% in the intervention group (teach-back), p=0.01<br><br>**2 EP:**<br>- Knowledge (Drugs: Prevalence of inhaler misuse for Discus) (30d): 80% in the control group vs. 25% in the intervention group, p=0.05<br>- Readmission (30d): 5/20 in the control group vs. 1/19 in the intervention group<br>- Death (30d): 3/20 in the control group vs. 0/19 in the intervention group	good
Sanchez Ulayar, A et al	Pharmaceutical intervention upon hospital discharge to strengthen understanding and adherence to pharmacological treatment	Farmacia Hospitalaria	2012	To determine the effectiveness of a pharmaceutical intervention with the patient upon hospital discharge and to improve understanding of pharmaceutical treatment and adherence to medication at home	Spain	Polymedicated medical inpatients, n=100	Single center RCT	**Intervention group** (n=50)<br>Pharmacist counseling and personalized medication plan	**Control group** (n=50)<br>Usual care	A pharmacist explained the drugs prescribed giving the patient a personalised medication timetable (with prescribed medication and when and which dose to take).The pharmacist explained why each drug had been prescribed, how to take it and why it was important to take the medication correctly.	**1EP:**<br>- Adherence (7d): 8 out of 41 patients in the control group vs. 29 out of 41 patients in the intervention group took all their medication in adherence to their prescription, p<0.001<br><br>**2EP:**<br>- Death (30d): 1/50 patient in the control group vs. 1/50 patient in the intervention group died<br>- Readmission (30d): 10/41 patients in the control group vs. 3/41 patients in the intervention group were readmitted to hospital (p<0.05)<br>**-** Readmission (60d): 13/41 patients in the control group vs. 3/41 patients in the intervention group were readmitted to hospital (p<0.01)	poor

© 2021 Becker C et al. *JAMA Network Open.*

Marušić, S et al	The effect of pharmacotherapeutic counseling on readmissions and emergency department visits	International journal of clinical pharmacy	2013	To evaluate the impact of pharmacotherapeutic counseling on the rates and causes of 30-day post-discharge hospital readmissions and emergency department visits	Croatia	Elderly medical patients (≥ 65 years) prescribed with ≥2 medications for chronic diseases n=160	Single center RCT	**Intervention group** (n=80)<br>Pre-discharge counseling by the clinical pharmacologist about each prescribed medication	**Control group** (n=80)<br>Usual care	Pre-discharge counseling (24h prior to discharge) by a specialist in clinical pharmacology<br>Information about each prescribed medication was given: indications for prescription, dosage and time of intake, importance of compliance, possible consequences of non-compliance, adverse drug reactions (ADR), prevention of ADRs, measures to be taken in case of ADRs	**1EP:**<br>- Readmission (30d): 5 patients in the control group vs. 6 patients in the intervention group were readmitted to hospital, p=0.754<br><br>**2 EP:**<br>- A&E Reattendance (30d): 22 patients in the control group vs. 14 patients in the intervention group visited an ED, p= 0.129<br>- Adherence (30d): 43 patients in the control group vs. 71 patients in the intervention group were compliant to their medication, p<0.001<br>- Adverse drug reactions (30d): 30 patients in the control group vs. 24 patients in the intervention group had ADRs, p=0.315<br>- Death (30d): 2 patients in the control group vs. 0 patients in the intervention group died	good

© 2021 Becker C et al. *JAMA Network Open.*

McCarthy, ML et al	Does providing prescription information or services improve medication adherence among patients discharged from the emergency department? A randomized controlled trial	Annals of emergency medicine	2013	Effect of prescription information on medication adherence	USA	Adult emergency patients with newly prescribed medication n= 3386	Multicenter RCT 4-group parallel design	**Intervention groups**<br>Group 1: practical information regarding prescription (n=971)<br>Group 2: MedlinePlus written information regarding drug information such as side effects, web-address with online access (n=991)<br>Group 3: combination of all information + phone number for additional information (n=991)	**Control group** (n = 987)<br>Usual care	Usual care included brief verbal instructions to patients about prescription medications at discharge<br><br>Practical prescription information / services offered information and assistance to reduce financial and logistic barriers related to filling a prescription (e.g., locating a convenient pharmacy)<br><br>MedlinePlus prescription information: comprehensive drug information (e.g., indication of drug, possible benefits and adverse effects,<br>Research assistants downloaded the drug information from MedlinePlus, a health information Web site, reviewed information with subjects and showed them the MedlinePlus Web site<br><br>Combination group was offered all of the information services offered to subjects randomized to the practical or MedlinePlus prescription information or services groups, plus they were given the telephone number of a clinical informationist to seek more information	**1EP:**<br>- Adherence to medication (7d): no significant difference in adherence between standard procedure and intervention groups (87% vs. 88%)<br><br>**2EP:**<br>- Incidents of adverse effects (7d): no meaningful difference between control and intervention groups (106/867 patients in the control group vs. 114/832 patients in the intervention group (group 3)<br>-Usefulness of drug information (7d): 474/864 patients in the control group vs. 526/832 patients in the intervention group found the information very useful<br>- Satisfaction with ED care (7d): 408/867 patients in the control group vs. 409/832 patients in group 3 were very satisfied with ED care<br>- A&E Reattendance (7d): no difference between control and intervention group 3 (71/867 patients in the control group vs. 85/832 patients in the intervention group)	good

© 2021 Becker C et al. *JAMA Network Open.*

Shah M et al	Diabetes transitional care from inpatient to outpatient setting: Pharmacist discharge counseling	Journal of Pharmacy Practice	2013	To evaluate the effect of inpatient pharmacist discharge counseling on outpatient diabetes medication adherence	USA	Inpatients with type 2 diabetes n=130	Single center RCT	**Intervention group** (n=64) Pharmacist discharge counseling	**Control group** (n=63) Usual care (routine diabetes education)	Pharmacist counseling prior to usual care and discharge on: medications dosing, benefits, refills, side effects importance of adherence, symptoms of hyper- and hypoglycemia, health eating, exercise, risk reduction for complications	**1EP:**<br>- Overall adherence (150d, PDC method): mean percentage of adherence to medication was 34.8% (37.9) in the control group vs. 55.2% (42.0), p=0.004<br><br>**2EP:**<br>- Adherence (30d): mean percentage of adherence was 44.1% (48.8) in the control group vs. 58.6% (48.4) in the intervention group, p=0.12<br>- Adherence (60d): mean percentage of adherence was 34.1% (45.9) in the control group vs. 52.7% (48.3) in the intervention group, p=0.16<br>- Adherence (90d): mean percentage of adherence was 36.4% (46.2) in the control group vs. 62.0% (48.2) in the intervention group, p=0.001<br>- Adherence (120d): mean percentage of adherence was 24.4% (41.6) in the control group vs. 47.2% (49.9) in the intervention group, p=0.006<br>- Death (30d): 1/64 in the control group and 1/65 in the intervention group died<br>- Adherence to follow-up appointment (90d): 43.9% in the control group vs. 60.5% in the intervention group, p=0.01	poor
de Oliveira-Filho, AD et al	Improving Post-Discharge medication adherence in patients with CVD: A pilot randomized trial	Arquivos brasileiros de cardiologia	2014	Effect of verbal and written information on medication adherence	Brazil	Cardiovascular patients n=61	Single center RCT	**Intervention group** (n=30) Drug counseling based on adherence questionnaire and written information	**Control group** (n=31) Usual care	Intervention group - MMAS 4 questionnaire and discharge counseling based on the questionnaire (information about disease, discharge medication, consequences of incompliance, side effects, dosage schedule) Evaluation of medication by pharmacist	**1EP:**<br>**-** Adherence to medication (30d): medication adherence improved from 41.9% to 48.4% in the control group vs. 58.1% to 83.3% in the intervention group, p=0.004<br><br>**2EP:**<br>- Medication adherence (1 year): 34.8% in the control group vs. 60.9% in the intervention group (p=0.203)<br>- Readmission (1 year): 15 (48%) in the control group vs. 6 patients (20%) in the intervention group, p=0.20<br>- Deaths (1 year): 6 (19%) in the control group vs. 3 (10%) in the intervention group	good

© 2021 Becker C et al. *JAMA Network Open.*

Moss, R et al	A nurse-led randomised controlled trial of a structured educational programme for patients starting warfarin therapy	Journal of Research in Nursing	2014	To investigate the effect of a nurse-led structured educational program on patient knowledge and satisfaction when starting anticoagulant therapy	UK	Patients commencing warfarin n=45	Single center RCT	**Intervention group** (n=21)<br>Structured counselling and educational video	**Control group** (n=24)<br>Usual care	Structured educational program including counselling of at least 30 minutes (counselling about the anticoagulant, mechanism, side effects and interactions, life style modifications, INR measurements, importance of compliance to the prescribed medication) and a 20 minute educational video about Warfarin of which a copy was given to the patients	**1EP:**<br>- Knowledge (Drug) at discharge: Mean score (out of 20) of 11.95 in the control group (n=21) vs. 19.08 in the intervention group (n=24), p<0.001<br>- Knowledge (Drug) at 90d: Mean score (out of 20) of 16.94 in the control group (n=18) vs. 18.38 in the intervention group (n=21), p=0.038<br><br>**2EP:**<br>- Adherence (180d): Time spent in the INR range was 41.1% in the control group vs. 56.7% in the intervention group, p=0.165	poor
Basger, B et al	Impact of an enhanced pharmacy discharge service on prescribing appropriateness criteria: a randomized controlled trial	International journal of clinical pharmacy	2015	To examine the effects of applying a validated prescribing appropriateness criteria-set during medication review of older patients at time of discharge	Australia	Elderly patients (≥ 65 years) with ≥5 medications n=216	Single center RCT	**Intervention group** (n=92)<br>Discharge medication counselling and medication review by clinical pharmacist	**Control group** (n=91)<br>Usual care	Medication counseling from the clinical pharmacist to facilitate completion of a medication review which was sent to their GP<br>Medication review: medication reconciliation, identification of potential causes of DRPs and recommendation for their resolution and prevention<br>Self-management was discussed, a copy of the review given to patients<br>Explanation of each discharge medication by a nurse (also in the control group)	**1EP:**<br>- Appropriateness criteria: No difference in criteria met between control and intervention group<br><br>**2EP:**<br>- Death (90d): 3/102 patients in the control group vs. 4/114 patients in the intervention group died<br>- Quality of life (SF-36): No difference in quality of life 90d after discharge	fair
Moore, SJ et al	Impact of video technology on efficiency of pharmacist-provided anticoagulation counseling and patient comprehension	The Annals of pharmacotherapy	2015	To evaluate differences in pharmacist time spent counseling and patient comprehension between informational videos and traditional face-to-face counseling	USA	Inpatients on oral anticoagulation (Warfarin) n=40	Single center RCT parallel-group	**Intervention group** (n=20)<br>Video counseling	**Control group** (n =20)<br>Oral counseling	Video-counseling: anticoagulation education via a prerecorded video provided on a tablet device<br><br>Oral counseling: face-to-face counseling<br><br>Both Group were asked questions utilizing the "teach-back method"	**1EP:**<br>- Pharmacist time spent counseling: overall pharmacist time was reduced in the video counseling group (P < 0.001)<br><br>**2EP:**<br>**-** Knowledge (Drug, at discharge): Post counseling OAK test scores did not differ between video and face-to-face counseling group (71.3% (95% CI: 66.3 to 76.1) in the control group (n=19) vs. 74.3 % (95% CI: 69.5 to 79.2) in the intervention group (n=19)<br>- Knowledge (Drug, 7d): 73.5% (27.1) in the control group (n=10) vs. 77.5% (14.4) in the intervention group (n=8)	poor

© 2021 Becker C et al. *JAMA Network Open.*

Olives, TD et al	Seventy-two-hour antibiotic retrieval from the ED: a randomized controlled trial of discharge instructional modality	The American journal of emergency medicine	2016	To examine the impact of instructional on 72-hour antibiotic retrieval among ED patients prescribed outpatient antibiotics for infections	USA	Emergency patients with acute infections and prescribed outpatient antibiotics n=2759	Single center RCT 3-group parallel design	**Group 1** (=826) Standard of care plus a brief text message containing antibiotic self-administration instructions<br><br>**Group 2** (n=810) Standard of care plus voicemail discharge instructions (antibiotic self-administration instructions)	**Control group** (=885) Standard of care discharge instructions	In addition to standard of care (routine verbal delivery of discharge instructions and a printed "after-visit summary"), group 1 received text messages from their treating physician with antibiotic self-administration instructions. Group 2 received voicemail discharge instructions containing spoken antibiotic self-administration instructions .	**1EP:**<br>- Successful retrieval of antibiotic prescription (72h): 756 (93.5%) of the control group vs. 682 (91.2%) in group 1 vs. 691 (94.0%) in group 2 retrieved their antibiotic prescription within 72 hours post discharge, p=0.078<br><br>**2EP:**<br>- Adherence (30d): 365/436 patients in the control group vs. 363/415 in group 1 vs. 343/417 in group 2 self-reportedly completed their antibiotic therapy<br>- Preference for discharge instruction modality	good
Press, VG et al	Effectiveness of Interventions to Teach Metered-Dose and Discus Inhaler Techniques. A Randomized Trial	Ann Am Thorac Soc	2016	Effects of two different educational strategies (teach-to-goal instruction vs. brief verbal instruction) in adults hospitalized with asthma or chronic obstructive pulmonary disease.	USA	Inpatients with Asthma or COPD n=120	Multicenter RCT	**Intervention group** (n=62) oral and written information regarding inhalers plus teach-to-goal	**Control group** (n = 58) oral and written information only	Patients in the intervention group received demonstration of correct use of inhaler, further evaluation of patients' technique, written information	**1EP:**<br>**-** Knowledge (Drugs: Prevalence of inhaler misuse) (30d): Misuse in intervention group (54%) was not significantly different from control group (70%) was not significantly different (p= 0.1)<br><br>**2EP:**<br>- Knowledge (Drugs: Prevalence of inhaler misuse) (90d): misuse in intervention group was significantly lower than control group (48% vs. 76%; p = 0.004)<br>- A&E reattendance: 9/54 in intervention vs. 16/53 in control group<br>- Readmission (30d): 6/54 in intervention vs 13/53 in control group	good

© 2021 Becker C et al. *JAMA Network Open.*

Sanii, Y et al	Role of pharmacist counseling in pharmacotherapy quality improvement	Journal of research in pharmacy practice	2016	Effect of patient counseling at discharge on treatment satisfaction and medication adherence	Iran	Inpatients in the respiratory ward n=200	Single center RCT	**Intervention group** (n=78)<br>Pharmacist counseling and education about prescribed medications	**Control group** (n=76)<br>Usual care	1. Patients were educated on and informed about health conditions and drug therapy (medication counseling on all prescribed medications), its side effects, inhaler technique assessment and education.<br>2. Comparison of discharge medication with preadmission regimens<br>3. Screening of previous drug-related problems (nonadherence, side effects)<br>4. Review of indications, directions for use, interactions, importance of adherence to medication, potential adverse effects	**1EP:**<br>- Adherence (30d): Adherence in the control group was 50.3% (27.1) vs. 93.2% (9.2) in the intervention group, p=0.010<br><br>**2EP:**<br>- Satisfaction (30d): 50.0 (16.2) in the control group vs. 83.5 (13.7) in the intervention group, p=0.012<br>- Readmission (30d): 8 patients in the control group vs. 0 patients in the intervention group were readmitted	poor
Biscaglia, S et al	A counseling program on nuisance bleeding improves quality of life in patients on dual antiplatelet therapy: A randomized controlled trial	PloS one	2017	The effect of a counseling program on the impact of nuisance bleeding on quality of life of patients on dual antiplatelet therapy	Italy	Inpatients on dual antiplatelet therapy, n=476	Single center RCT	**Intervention group** (n=224)<br>Counseling program focused on nuisance bleeding	**Control group** (n =224)<br>Usual care	15-minutes meeting assessment of risk for bleeding, advantages side effects and importance of adherence to DAPT explained<br><br>Patients received a brochure describing DAPT advantages, side effects and management	**1EP:**<br>**-** Quality of life 30d after discharge (EuroQol): Quality of life significantly higher in intervention than in control group (73 in the control group vs. 81 in the intervention group, p<0.001)<br><br>**2EP**<br>- Quality of life 6 months after discharge (EuroQol): Quality of life significantly higher in intervention group (74 in the control group vs. 82 in the intervention group, p<0.001)<br>- A&E reattendance (180d): 6/224 in the control group vs. 4/224 in the intervention group, p=0.5<br>- Readmission (180d): 4/224 in the control group vs. 3/224 in the intervention group, p=0.7<br>- Death (180d): 2/224 in the control group and 1/224 in the intervention group, p=0.6	good

© 2021 Becker C et al. *JAMA Network Open.*

Castelli, MR et al	Effect of a Rivaroxaban Patient Assistance Kit (R-PAK) for Patients Discharged With Rivaroxaban: A Randomized Controlled Trial	Hospital pharmacy	2017	The effect of the Rivaroxaban Patient Assistance Kit (R-PAK) as a counseling strategy on medication adherence	USA	Patients with venous thromboembolism n=25	Single center RCT	**Intervention group** (n=12)<br>Rivaroxaban Patient Assistance Kit	**Control group** (n =13)<br>Usual care	Rivaroxaban Patient Assistance Kit: includes a reminder card stating the dates of dose transition and a customizable pillbox as well as an educational handout describing adverse effects, administration, and monitoring<br>Patients were taught how to properly fill and utilize the pillbox.<br><br>All patients were educated on the indication, dosing regimen, adverse effects, and monitoring required for rivaroxaban	**1EP:**<br>- Percentage of patients who properly transitioned to Rivaroxaban 20 mg once daily on day 22: no significant difference between intervention and control group (67% vs. 69%, p=0.89)<br><br>**2EP:**<br>- Adherence to medication (30d): 97.65% in the control group vs. 99.8% in the intervention group, p=0.07<br>- Adherence (30d): 12/13 patients in the control group vs. 12/12 in the intervention group showed ≥90% adherence, p=0.33<br>- Death (30d): no deaths in control/intervention group, p=1<br>- Self reported side effects (30d): 3/13 patients in the control group vs. 0/12 patients in the intervention group, p=0.08	poor
Chakravarthy, B et al	Randomized pilot trial measuring knowledge acquisition of opioid education in emergency department patients using a novel media platform	Substance Abuse	2017	To determine if an educational intervention via a brief video discharge instruction leads to higher patient knowledge than the standard of care	USA	Pain patients with outpatient opioid prescription n=52	Single center RCT	**Intervention group** (n=25)<br>6-minute video on proper usage of opioids in addition to standard care	**Control group** (n=27)<br>Usual care (verbal instruction, informational sheet)	Animated 6-minute discharge video on opioid safety, proper usage, storage and disposal in addition to standard discharge procedures (nurse led on side effects, cautions against concomitant use of opioids)	**1EP:**<br>- Knowledge (Drug, at discharge): the control group showed 65% knowledge acquisition (16.8/26 points, SD=4.53) vs. 82% in the intervention group (21.2/26 points, SD=4.98), p<0.001	poor
Al-Hashar, A et al	Impact of medication reconciliation and review and counselling, on adverse drug events and healthcare resource use	International journal of clinical pharmacy	2018	Impact of medication reconciliation and counselling intervention on adverse drug events after discharge	Oman	Medical inpatients n=587	Single center RCT	**Intervention group** (n=286)<br>Medication reconciliation intervention	**Control group** (n=301)<br>Usual care	Involvement of pharmacist to<br>1) Detect discrepancies and resolve them<br>2) Bedside counseling regarding medication<br>3) Medication list with educational material	**1EP:**<br>- Amount of preventable adverse drug events 30d after discharge: 59 in the control group vs. 27 ADEs in intervention group, p=0.008<br><br>**2EP:**<br>- Readmission (30d): 44/301 in the control group vs. 39/286 in intervention group, p=0.907<br>- A&E reattendance (30d): 59/301 in the control group vs. 46/286 in intervention group, p=0.344<br>- Death (30d): 6/301 in the control group vs. 7/286 in the intervention group, p=0.784	good

© 2021 Becker C et al. *JAMA Network Open.*

Maruši ć, S et al	Impact of pharmacotherapeutic education on medication adherence and adverse outcomes in patients with type 2 diabetes mellitus: A prospective, randomized study	Croatian medical journal	2018	To evaluate the impact of pharmacotherapeutic education on 30-day post-discharge medication adherence and adverse outcomes in patients with type 2 diabetes mellitus	Croatia	Patients with Type 2 Diabetes n=130	Single center RCT	**Intervention group** (n=65)<br>Individual pre-discharge pharmacotherapeutic education	**Control group** (n =65)<br>Usual care	Both groups during the hospital stay received standardized diabetes education<br><br>Intervention group received additional individual predischarge pharmacotherapeutic education about the discharge prescriptions; sessions took 30-minutes sessions, conducted by a physician, patients received information regarding indications for medication, dosage and administration time, the importance of medication adherence, possible consequences of non-adherence, possible ADRs, prevention and early detection of ADRs, and measures to be taken if an ADR is suspected. All patients were given a leaflet containing the same information in writing.	**1EP:**<br>- Adherence to medication (30d): 41/61 patients in the control group vs. 57/64 patients in the intervention group were adherent, p=0.003<br><br>**2EP:**<br>- Adverse outcome: no significant difference regarding adverse outcome between control and intervention group (36/61 in the control group vs. 31/64 in the intervention group, p=0.236)<br>- Readmission (30d): 8/61 patients in the control group vs. 5/64 in the intervention group, p=0.332<br>- A&E Reattendance (30d): 15/61 patients in the control group vs. 14/64 in the intervention group, p=0.719<br>- Death (30d): 3/61 patients in the control group vs. 1/64 in the intervention group, p=0.357<br>- Adverse drug reactions (30d): 25/61 in the control group vs. 23/64 in the intervention group	poor
Graabaek, T et al	Effect of a medicines management model on medication-related readmissions in older patients admitted to a medical acute admission unit-A randomized controlled trial	J Eval Clin Pract	2019	Effect of a pharmacist-led medicines management model among older patients on medication-related readmissions	Denmark	Medical inpatients older than 65 n=600	Single center RCT<br>3-group parallel design	**ED group - basic intervention** (n=200)<br><br>**Stay group - extended intervention** (n=200)	**Control group** (n=200)<br>Standard discharge procedure	Basic intervention: pharmacist-led medication review (including patient interview and medication reconciliation)<br><br>Extended intervention: pharmacist-led medication review (including patient interview and medication reconciliation)<br>Patient counselling and a medication report at discharge	**1EP:**<br>- Medication-related readmission within 30 d after discharge: 11 control patients, 9 ED patients, and 5 STAY patients had a medication-related readmission<br><br>**2EP:**<br>- Death (30d): 2/200 patients in the control group vs. 1/200 in the intervention group died, p=0.603<br>- Overall mortality (180d): no significant difference (16/200 in the control group vs. 13/200 in Stay group, p=0.601)<br>- Overall readmission rate (30d): no significant difference regarding readmission between the control group (67/198) vs. Stay group (46/194), p=0.086<br>- Overall readmission rate (180d): 1/184 patients in the control group vs. 0/187 in the intervention group were readmitted, p=0.245<br>- A&E Reattendance (180d): 0/184 in the control group vs. 0/187 in the intervention group, p=0.866	good

© 2021 Becker C et al. *JAMA Network Open.*

Yin, D et al	The effect of inpatient pharmaceutical care on nephrotic syndrome patients after discharge: a randomized controlled trial	International journal of clinical pharmacy	2020	To evaluate the impact of pharmacist counseling on medication adherence and other patient clinical outcomes.	China	Inpatients with nephrotic syndrome n=61	Single center RCT	**Intervention group** (n=31)<br>Pharmacist-delivered intervention	**Control group** (n=30)<br>Usual care	Medication reconciliation before discharge, patient education during hospitalization, discharge counseling and education. Patients received verbal and education by teach-back method including medication information, lifestyle and diet	1EP:<br>medication adherence 30 days after discharge: no significant difference, 16 patients (51.6%) in the intervention group and 11 patients (36.7%) in the control group showed high adherence, p= 0.306<br><br>2EP:<br>medication adherence 90 days after discharge: no significant difference between intervention and control group, p=0.120<br><br>medication adherence 180 days after discharger: patients in the intervention group showed higher rates of adherence than patients in the control group, (45.2% with high adherence in intervention vs.16.6% in control group, p= 0.026)<br><br>Planned return visit 30 days after discharge: 31 (100%) in intervention vs. 22 (73.3%) in control group, p= 0.002<br><br>readmission 180 days after discharge: no significant difference between intervention and control group (5/31 vs. 7/30, p= 0.449)	poor
Interventions: Education regarding disease and its management												
Waggoner, DM et al	Physician influence on patient compliance: a clinical trial	Annals of emergency medicine	1981	To measure the effect of altering three possible impediments to care provided patients with non-emergency problems	USA	Emergency patients with symptomatic urinary tract infections n=89	Single center RCT	**Intervention group** (n=46)<br>Oral information by the physician about illness and medication	**Control group** (n=43)<br>Usual care	Extra time spent with a senior physician: education about illness, medication and need to return, bypass of the usual clerical procedures at discharge, promise of continuity of care and no waiting at follow-up	**1EP:**<br>- Adherence (return rate for follow-up, 3 weeks): in control group 14 (37.6%) vs. 26 (56.5%) in the intervention group	poor
Ben Said, M et al	A comparative study between a computer-aided education (ISIS) and habitual education techniques for hypertensive patients	Proceedings Symposium on Computer Applications in Medical Care	1994	Effect of using a computer-aided education program as a complement to the existing patient education methods on patient knowledge about hypertension	France	Hospitalized patients with hypertension n=158	Single center RCT	**Intervention group** (n = 69)<br>30 to 60 minute educational session using a computer program in addition to usual care	**Control group** (n = 69)<br>Usual care (educational sessions, dialogs with physicians, nurses, dieticians and brochures, videotapes, posters)	Educational computer program about hypertension to be used without the educator full-time assistance (topics: arterial pressure, epidemiology, heart, atherosclerosis, risk factors and treatment)	**1EP:**<br>- Knowledge (Diag, 60d): 2.4(3.2) points increase of health knowledge compared to baseline in the control group vs. 3.8(3.6) points in the intervention group, p = 0.02	poor

© 2021 Becker C et al. *JAMA Network Open.*

Hayes, KS et al	Randomized trial of geragogy-based medication instruction in the emergency department	Nurs Res	1998	To asses the effect of individualized computer generated discharge instructions on patients' knowledge	USA	Elderly emergency patients n=60	Multicenter RCT	**Intervention group** (n=30) Individualized elderly-friendly written information	**Control group** (n = 30) Usual care	Computer-generated individualized information including a brief description of diagnosis, self-care management of condition, discharge medications and follow-up physician instruction	**1EP:** **-** Knowledge of Medication Subtest (KMS, 72h): 52 (SD = 7.93) in the control group vs. 47.55 (SD = 7.78) in the intervention group (higher scores reflect less knowledge)	poor
Davies, M et al	Evaluation of a hospital diabetes specialist nursing service: a randomized controlled trial	Diabetic medicine: a journal of the British Diabetic Association	2001	To evaluate the effectiveness and cost implications of a hospital diabetes special nursing service	UK	Patients with either Type 1 or Type 2 diabetes n=300	Multicenter RCT	**Intervention group** (n=148) Care and advice from a diabetes specialist nurse (DSN)	**Control group** (n=152) Usual care	DSN care from arrival until discharge from the ward: structured patient education and practical management advice (verbal and written case-note feedback to ward-based medical and nursing staff)	**1EP:** - Readmission (1 year): 38 (25.0%) readmissions in the control group vs. 37 (25.0%) readmissions in the intervention group, p=1.0 **2EP:** - Satisfaction with care (1 week post-study): 59% in the control group vs. 91% in the intervention group, p<0.001 - Knowledge (diagnosis, 1 week post-study): 48% in control group vs. 74% in intervention group, p<0.05 - Quality of life (ADDQoL, post-study): 0.40 in control group vs. 0.88 in intervention group, p>0.05	poor

© 2021 Becker C et al. *JAMA Network Open.*

Morice, AH. et al	The role of the asthma nurse in treatment compliance and self-management following hospital admission	Respiratory medicine	2001	To determine if asthma nurse intervention during hospital admission increases knowledge and improves self-management and if it impacts hospital-readmissions and emergency call-outs of GPs	UK	Patients with acute asthma n=80	Single center RCT	**Intervention group** (n=40)<br>Individualized educational sessions with an asthma nurse until hospital discharge	**Control group** (n=40)<br>Usual care	At least two individualized educational sessions by an asthma nurse (discussion on basic facts about asthma, possible lifestyle changes, need and use of medication, booklet about asthma therapy). An individualized self-management plan was determined with written instructions and patients received a peak flow meter to take home (with instructions and guidelines when to seek emergency care)	**1EP:**<br>- Self-management: knowledge and use of medication such as inhalers, peak flow meters, knowledge of asthma, actions taken in case of worsening of symptoms at six weeks and six months post discharge (all p<0.01)<br><br>**2EP:**<br>- Readmission (1 year): 11 patients in the control group vs. 10 patients in the intervention group were readmitted<br>- A&E Reattendance (1 year): 0 patients in the control group vs. 2 patients in the intervention group	poor
Osman , LM et al	A randomised trial of self-management planning for adult patients admitted to hospital with acute asthma	Thorax	2002	To determine if a brief self-management programme given during hospital admission reduces readmission	UK	Patients with acute asthma n=280	Single center RCT	**Intervention group** (n=135)<br>Self-management programme with an educational session and a written self-management plan	**Control group** (n=145)<br>Usual care	Structured and educational self-management programme by a trained respiratory nurse on two occasions during hospital stay regarding knowledge about asthma, methods to recognize and avoid risk factors and basic information about medication<br><br>Booklet<br><br>Written self-management plan (symptom and peak flow based) based on discharge medication for the immediate time after discharge	**1EP:**<br>- Readmission (1 year): 27% (38/140) (of control group vs. 17% (22/131) of intervention group were readmitted, p=0.04<br><br>**2EP:**<br>- Readmission (30d): 4 patients of the control group vs. 1 patient in the intervention group were readmitted, p=0.4<br>- Patient morbidity (30d): Patients in the intervention group were more likely than control group patients to report no daytime wheeze (OR2.6, 95% CI: 1.5 to 5.3), no night disturbance (OR 2.0, 95% CI 1.2 to 3.5) and no activity limitation (OR 1.5, 95% CI 0.9 to 2.7)<br>- Patient satisfaction with explanation (30d): 76% (89/118) of the control group vs. 100% (108/108) of the intervention group were satisfied, p=0.000	good

© 2021 Becker C et al. *JAMA Network Open.*

Gwadry-Sridhar, FH et al	Pilot study to determine the impact of a multidisciplinary educational intervention in patients hospitalized with heart failure	American heart journal	2005	To examine the impact of a compliance enhancing intervention on medication compliance and morbidity in heart failure	Canada	Patients with heart failure and a LVEF < 40%<br>n=134	Single center RCT	**Intervention group** (n=66)<br>Booklets, video and education by a multidisciplinary team	**Control group** (n=68)<br>Usual care (booklets and video)	2 booklets and video-teaching about congestive heart failure were provided to the patient.<br>2.5 hours of education over two days, ending with discharge.<br>Education about compliant medication use as well as diet and lifestyle modifications were provided by a multidisciplinary team (nurse or educator and hospital pharmacist).<br>Education techniques consisted of personalized feed-back, oral, written and visual props and media videos.	**1EP:**<br>Knowledge (Diag, 1 year), Knowledge acquisition questionnaire (KAQ): Mean change in knowledge score of 1.38 (2.16) in control group vs. 2.24 (2.46) in the intervention group<br><br>**2EP:**<br>- Adherence: no significant difference to be seen in the RR for noncompliance in the control group vs. intervention group<br>- Quality of life (MLHFQ, 1 year): 32.19 in the control group vs. 25.75 in the intervention group<br>- Quality of life (SF-36, PCS): 37.38 in the control group vs. 37.15 in the intervention group, p=0.92<br>- Quality of life (SF-36, MCS): 51.94 in the intervention group vs. 52.38 in the control group, p=0.74	good

© 2021 Becker C et al. *JAMA Network Open.*

Koellin g, TM et al	Discharge education improves clinical outcomes in patients with chronic heart failure	Circulation	2005	To examine if a patient-targeted heart failure education program at hospital discharge improves clinical outcomes in patients with chronic heart failure	USA	Patients with heart failure and a LVEF < 40%<br>n=223	Single center RCT	**Intervention group** (n=107)<br>Standard discharge information plus patient-targeted heart failure education	**Control group** (n=116)<br>Usual care (written discharge information about medications, side-effects, dietary and activity instructions, description of heart failure symptoms and when to call a physician)	60 minute one-on-one teaching with a nurse educator before discharge about heart-failure specific information (intravascular volume overload) and pharmaceutical therapies (mechanisms of diuretics), dietary restrictions, self-care behaviors (daily weight monitoring, smoking cessation, avoidance of noxa, what to do with worsening symptoms)<br><br>Treatment guidelines for heart failure in layman's terms	**1EP:**<br>- Numbers of days hospitalized and/or dead after 180d: 2103 days (mean 18+/-37 days) in the control group vs. 1554 days (mean 14+/-36 days) in the intervention group<br><br>**2EP:**<br>-Readmission (180d): 54 patients in the control group vs. 34 patients in the intervention group were readmitted to hospital, RR 0.59 (95% CI: 0.38 to 0.91), p=0.014<br>- Death (180d): 10 patients (8.6%) in the control group vs. 7 (6.5%) in the intervention group died, RR 0.94 (95% CI: 0.34 to 2.6), p=0.91<br>- Quality of life (MLHF, range 0–105, from best to worst), 30d: 42 (25) in the control group vs. 38 (22) in the intervention group, p=0.049<br>-Quality of life (MHLF), 3 months: 42 (25) in the control group vs. 41 (22) in the intervention group<br>- Self-care behavior (30d): self-care practices score (of total 6) in the control group 3.0 (1.5) vs. 3.6 (1.5) in the intervention group, p=0.001<br>- Costs of care (180d): $8292 (+/- 11299) for control subjects vs. $5369 (+/- 9096) for intervention subjects<br>- Time to death or first hospitalization was significantly longer for the education group (p=0.012)	fair

© 2021 Becker C et al. *JAMA Network Open.*

Koonce, TY et al	A pilot study to evaluate learning style-tailored information prescriptions for hypertensive emergency department patients	Journal of the Medical Library Association : JMLA	2011	To evaluate if learning style-tailored education materials are effective in increasing hypertension knowledge in emergency room patients	USA	Emergency patients with hypertension n=76	Single center RCT	**Intervention group** (n=31)<br>Discharge instructions and information prescription tailored to patients' specific learning-style preferences	**Control group** (n=31)<br>Usual care (printed instruction sheet)	Information prescription tailored to the patient's individual learning style: visual learners (handout with graphic images), read/write learners (structured text), aural learners (podcast) and kinesthetic learners (interactive web-based application). Each learning style was provided with information about an overview on hypertension, risk factors and prevention and treatment options such as lifestyle changes. During the learning process study personnel remained present.	**1EP:**<br>- Knowledge (Diagnosis, 14d): Knowledge scores did not differ between control and intervention group (75.3 out of 100 in the control group vs. 71.2 in the intervention group)<br><br>**2EP:**<br>- Satisfaction (14d)**:** 26/31 patients in the control group vs. 29/31 patients in the intervention group were satisfied<br>- Ability to understand the information (14d): 26/31 in the control group vs. 27/31 in the intervention group found the instructions easy to understand	fair
Giuse, NB et al	Using health literacy and learning style preferences to optimize the delivery of health information	Journal of Health Communication	2012	To assess health literacy and learning preferences	USA	Emergency patients with high blood pressure n= 196	Single center RCT<br>Two experiments	**Intervention group 1** (n=40)<br>Standard of care discharge instructions and educational high blood pressure material adapted to patients' health literacy level<br><br>**Intervention group 2** (n=46)<br>Standard of care discharge instructions and educational high blood pressure material adapted to patients' health literacy levels and learning style preference	**Control group 1** (n=45)<br>Standard of care discharge instructions<br><br>**Control group 2** (n=41)<br>Standard of care discharge instructions	**Intervention 1:** In addition to standard of care discharge instructions, patients were given personalized hypertension material containing minimal information needed to correctly answer the hypertension knowledge test. Patients with low health literacy received an additional set. The information was in written in understandable language.<br><br>**Experiment 2:** The personalized hypertension material was adapted to patients' learning styles: illustrated handouts (visual learners), written information (read/write learners), audio version and CD (aural learners), card-sorting activity (kinesthetic), all formats (multimodal learners)	**Experiment 1:**<br>**1EP:** Knowledge (Diagnosis, 14d): Patients in the control group answered 7.6/17 questions correctly vs. 10.9 correct answers in the intervention group<br><br>**Experiment 2:**<br>**1EP:** Knowledge (Diagnosis, 14d): Patients in the control group answered 8.9 questions correctly vs. 14.2 in the intervention group	poor

© 2021 Becker C et al. *JAMA Network Open.*

Kommuri, NVA et al	Relationship between improvements in heart failure patient disease specific knowledge and clinical events as part of a randomized controlled trial	Patient Education and Counseling	2012	To examine the changes in performance on heart failure knowledge assessments administered before and after discharge education	USA	Patients with heart failure and a LVEF < 40%, n=265	Single center RCT	**Intervention group** (n=137)<br>1h long nurse educator delivering heart failure education program	**Control group** (n=128)<br>Usual care	1h long heart failure education program by a nurse educator about the basic principles of heart failure, the role of dietary sodium, the importance of limitation of fluid intake, the mechanisms of diuretics and a rationale for other pharmacotherapy.<br>Specific instructions were given to limit the sodium intake to 2000mg/d and the fluid intake to 2000ml/d.<br>Information about the importance of daily weight monitoring, self care behaviors, compliance to medication, smoking cessation, avoiding NSAR, limitation of noxa and measures to take when symptoms are worse<br>In addition, written information was given to the intervention group.	**1 EP:**<br>- Knowledge (Diagnosis, 90d): patients in the control group scored 9/30 points (HFKQ) vs. 11/30 points in the intervention group	fair
Perera, K et al	Medium of language in discharge summaries: Would the use of native language improve patients' knowledge of their illness and medications? Journal of Health Communication	J Health Commun	2012	To investigate if the use of native language improves patients' knowledge of their illness and medication	Sri Lanka	Patients with newly diagnosed noncommunicable chronic diseases (excluding malignancies) n=130	Single center RCT	**Intervention group** (n=65)<br>English discharge summary and supplementary discharge summary (native language)	**Control group** (n=65)<br>Usual care (customary discharge summary in English)	In addition to the customary English discharge summary, a supplementary discharge summary was given to patients. The supplementary summary had the diagnosis and prescribed medication written in patients' native language (Sinhala, Tamil)	**1EP:**<br>- Knowledge (Diagnosis, 14d): Of a total score of 100 points, the control group achieved 27.95 (41.26) points vs. 81.41 (34.64) points in the intervention group, p<0.001<br><br>**2EP:**<br>- Knowledge (Drug, 14d): Of a total score of 100 points, the control group achieved 12.56 (20.44) points vs. 54.48 (33.92) points in the intervention group, p<0.001	good

© 2021 Becker C et al. *JAMA Network Open.*

Lin, R et al	Effect of a patient-directed discharge letter on patient understanding of their hospitalisation	Internal medicine journal	2014	Effect of a patient-directed discharge letter on patients' understanding	Australia	Medical inpatients n=67	Single center RCT	**Intervention group** (n=32)<br>Patient-directed discharge letter	**Control group** (n=35)<br>Usual care	**Pa**tient-**d**irected **d**ischarge **le**tter (PADDLE) written by treating physician<br>(1) reason for hospitalisation, (2) the tests performed and their results, (3) treatments received (4) recommendations for following discharge	**1EP:**<br>- Patients' understanding: intervention participants increased their scores in all four domains between baseline and post-intervention, no significant difference between control and intervention group regarding knowledge after 3 and 6 months<br><br>**2EP:**<br>- Readmission 6 months after discharge: no difference between control (20%) and intervention group (21%) with regard to readmission	poor
Fuenzalida, C et al	Nurse-led educational intervention in patients with atrial fibrillation discharged from the emergency department reduces complications and short-term admissions	Emergencias	2015	To assess if a nurse-led education for patients with atrial fibrillation discharged from the emergency department improves the patients' understanding of arrythmia and its treatment and reduces the number of complications and arrythmia-related admissions	Spain	Emergency patients with atrial fibrillation (AF) n=240	Single center RCT	**Intervention group** (n=116)<br>Nurse-led education and information leaflet about AF, its treatment, precautions to take, warning signs and pulse taking	**Control group** (n=124)<br>Usual care	Nurse-led patient education about the basic aspects of arrhythmia, possible complications, its treatment, precautions to take and alarming symptoms.<br>Instructions on how to take pulse manually and to do so at least once a week<br>Advice to visit their GP<br>Personalized leaflet with information about the prescribed medication and a summary of the previously described information	**1EP:**<br>- Combined (Death and Complications): 30 patients in the control group vs. 16 patients in the intervention group, p=0.04<br><br>**2EP:**<br>- Knowledge (Diag, 30d): overall, no significant difference between the two groups could be shown<br>- Death (90d): 9/124 patients in the control group vs. 6/116 patients in the intervention group died<br>- Readmission (30d): 15/124 patients in the control group vs. 8/116 patients in the intervention group were readmitted<br>- Readmission (90d): 26/124 patients in the control group vs. 13/116 patients in the intervention group were readmitted, p=0.041	fair
Adamuz, J et al	Impact of an Educational Program to Reduce Healthcare Resources in Community-Acquired Pneumonia: The EDUCAP Randomized Controlled Trial	PloS one	2015	Effect of an educational program for inpatients on healthcare uitlization after discharge	Spain	Medical inpatients with community-acquired pneumonia n=207	Multicenter RCT	**Intervention group** (n=102)<br>Education at discharge regarding CAP	**Control group** (n =105)<br>Usual care	Educational program conducted by nurses between 24-72h before discharge regarding fluid intake, medication adherence, vaccination, knowledge and management of disease<br>Two sessions of 30 minutes each<br>Patients also received handout about self-management of CAP	**1EP:**<br>- Intervention significantly reduced healthcare utilization within 30d after discharge<br>- A&E reattendance (30d): 27/105 in control vs. 11/102 in intervention group, p=0.007<br>- Readmission to hospital (30d): 18/105 in control vs. 5/102 in the intervention group, p=0.007<br><br>**2EP:**<br>- Satisfaction (30d): 19/105 patients in the control group vs. 84/102 patients in the intervention group were satisfied, p<0.001<br>- Knowledge (Diag, 30d): 21/105 patients in the control group vs. 100/102 patients in the intervention group had knowledge regarding their disease and management 30d after discharge, p<0.001<br>- Adherence (30d): 101/105 in the control group vs. 98/102 in the intervention group were adherent, no difference regarding adherence to medication between control and intervention group, p=1	good

© 2021 Becker C et al. *JAMA Network Open.*

Chan, H-Y et al	Evaluation of a tablet-based instruction of breathing technique in patients with COPD	International journal of medical informatics	2016	Effect of using a tablet computer with the Breathing Easier Support Toolkit (BEST) to instruct and assist COPD patients during the process of respiratory retraining	Taiwan	Patients with COPD (FEV1/FVC < 0.7) n=71	Single center RCT	**Intervention group** (n=36) Teaching about PLB (pursed lip breathing) using tablet computers	**Control group** (n=35) Teaching about PLB (pursed lip breathing) in traditional setting	Three teaching units at bedside or in the classroom (for both groups). BEST (tablet application to assist with the instruction of the PLB technique in a breathing retaining program) was used only to teach patients in the intervention group. The teaching included several sessions about PLB to learn its technique.	**1EP:**<br>- Self-efficacy of breathing technique (30d): mean score of 40.6 (9.1) in the control group vs. 42.0 (7.7) in the intervention group<br>- Self-efficacy of breathing technique (90d): mean score of 45.6 (8.0) in the intervention group vs. 43.2 (9.7) in the intervention group<br><br>**2EP:**<br>- Correct breathing technique (30d): mean score of 23.1 (4.6) in the control group vs. 24.5 (3.3) in the intervention group<br>- Correct breathing technique (90d): mean score of 24.3 (3.3) in the control group vs. 23.1 (4.0) in the intervention group<br>- Quality of life (30d): 12.8 (8.0) in the control group vs. 12.3 (7.6) in the intervention group (CAT, max. = 40 points, higher score = poorer QoL)<br>- Quality of life (90d): 10.1 (5.8) in the control group vs. 11.9 /7.1) in the intervention group<br>- Death (30d): 4/35 patients in the control group vs. 3/36 in the intervention group died<br>- Death (90d): 6/35 patients in the control group vs. 5/36 in the intervention group died	poor
Hill, B et al	Automated pictographic illustration of discharge instructions with Glyph: impact on patient recall and satisfaction	Journal of the American Medical Informatics Association : JAMIA	2016	Effect of pictograph-enhanced discharge instructions on patients' recall of and satisfaction with their discharge instructions.	USA	Cardiovascular inpatients n=144	Single center RCT	**Intervention group** (n=71) Pictograph-enhanced discharge instructions	**Control group** (n =73) Standard discharge procedure	Discharge education by nurses Instruction handouts were enhanced by pictures to illustrate information	**1EP:**<br>- Post-teaching recall of instructions: no difference between the two groups, p=0.852<br><br>**2EP**<br>**-** Satisfaction (7d): 92% in the control group vs. 97% in the intervention group were satisfied with the amount of information, p=0.142	poor

© 2021 Becker C et al. *JAMA Network Open.*

Kato, NP et al	How effective is an in-hospital heart failure self-care program in a Japanese setting? Lessons from a randomized controlled pilot study	Patient preference and adherence	2016	Effect of a heart-failure teaching on self-care and knowledge	Japan	Inpatients with heart failure<br>n=32	Single center RCT	**Intervention group** (n=16)<br>Self-care education	**Control group** (n =73)<br>Standard discharge procedure	Multidisciplinary face-to-face counselling by dietician, pharmacist and nurses regarding illness, risk factors, red flags and healthy lifestyle<br>Mean education time 68 minutes	**1EP:**<br>- HF self-care behavior (EHFScBS): no significant differences int he EHFScBS scores between control and intervention groups after 6 months, p=0.65<br><br>**2EP:**<br>- HF Knowledge (Diag, 30d): knowledge score in the control group was significantly lower than that in the intervention care group at 1 month after discharge (8.7±4.8 vs. 13.1±1.7; p=0.03)<br>- Time to first readmission: significant benefit of the HF program on time to the first HF hospitalization, p =0.04<br>- Death (6 months): 5/15 in the control group vs. 1/14 in the intervention group died, p=0.04<br>- Death (1 year): 7/15 in the control group vs. 2/14 in the intervention group died, p=0.04	fair
Fuenzalida, C et al	Long-term benefits of education by emergency care nurses at discharge of patients with atrial fibrillation	International Emergency Nursing	2017	To assess if an educational nursing intervention at discharge from the emergency room (ER) had a long-term effect in patients with atrial fibrillation (AF)	Spain	Emergency patients with atrial fibrillation (AF)<br>n=240	Single center RCT	**Intervention group** (n=116)<br>Basic explanation about arrythmia and its treatment, precautions and warning signs, a training to take their pulse, information leaflet	**Control group** (n=124)<br>Usual care	Nurse-led patient education about the basic aspects of arrhythmia, possible complications, its treatment, precautions to take and alarming symptoms.<br>Instructions on how to take pulse manually and to do so at least once a week<br>Advice to visit their GP<br>Personalized leaflet with information about the prescribed medication and a summary of the previously described information	**1EP:**<br>- Combined (Death and Complications, 1 year): 60 patients in the control group vs. 37 patients in the intervention group, p=0.005<br><br>**2EP:**<br>- A&E Reattendance (1 year): 65 patients in the control group vs. 51 patients in the intervention group, OR=0.676 (95% CI: 0.405-1.128), p=0.134<br>- Readmission (1 year): 43 in the control group vs. 31 in the intervention group, OR=0.662 (95% CI: 0.380-1.152), p=0.144<br>- Adverse drug reactions (1 year): whether antiarrhythmic treatment-related nor anticoagulant-related complications did reach significant differences between the two groups (p=0.744 or 0.909)<br>- Complications (1 year): Heart failure as a complication of AF was significantly higher in the control group (33/124) vs. in the intervention group (19/116), p=0.045. Other complications (Stroke or systemic embolism) did not reach significant differences between the two groups<br>- Death (1 year): 43 patients in the control group vs. 26 patients in the intervention group died	fair

© 2021 Becker C et al. *JAMA Network Open.*

Athar, MW et al	The Effect of a Personalized Approach to Patient Education on Heart Failure Self-Management	Journal of personalized medicine	2018	Effect of image of inferior vena cava (IVC) as personalized education approach on medication adherence	USA	Inpatients with decompensated heart failure n=97	Single center RCT	**Intervention group** (n=50) Education and image of IVC	**Control group** (n =47) Usual care (only generic information)	Intervention group patients were shown their IVC images by the ultrasonographer, who also provided them with real-time scripted educational information information was tailored to the amount of distension of IVC Patients in intervention group also received laminated Patient Education Tool	**1 EP:**<br>- Adherence to HF regimen (MOSSAS-3HF) (30d): adherence to HF treatment not different between control and intervention group (11.7 ± 3.0 vs. 11.8 ± 2.8, p = 0.90)<br><br>**2EP:**<br>- Readmission (30d): no difference between control and intervention group (7/44 vs. 7/46), p=0.93<br>- A&E reattendance (30d): no difference between control and intervention group (7/44 vs. 8/46), p=0.85	good
Breathett, K et al	Pilot randomized controlled trial to reduce readmission for heart failure using novel tablet and nurse practitioner education	Journal of Heart and Lung Transplantation	2018	Effect of tablet application for education on readmission rates	USA	Inpatients with heart failure n=126	Single center RCT	**Intervention group** (n=60) Education by Nurse practitioner enhanced by tablet application	**Control group** (n =66) Standard discharge with nurse practitioner	Education included one-on-one discussion of heart failure materials<br><br>Tablet application was an interactive audio-visual program, which provides individualized education and flagged patient questions to medical staff.<br>Application had four specific topics: heart failure overview, nutrition plan, importance of medication adherence and lifestyle modification	**1EP:**<br>- Readmission (30d): readmission tended to be lower in intervention group with 16/60 in the control group and 7/53 in the intervention group readmitted, p=0.08<br><br>**2EP:**<br>- Patient satisfaction (VAS 0-10, 30d): median 10 in the intervention group vs. 8.3 in the control group<br>- Self-perceived knowledge of purpose of medication (Drug, 30d): 80.0 % in the control group vs. 83.3% in the intervention group reported to know the purpose of medication, p=0.70<br>- Self-perceived knowledge regarding meaning of heart failure (Diag): 70% in the control group vs. 78.6% in the intervention group indicated to know the meaning of heart failure, p=0.37	poor
Jasinski, MJ et al	Family consultation to reduce early hospital readmissions among patients with end-Stage renal disease: A randomized clinical trial	Clin J Am Soc Nephrol	2018	Effect of education of patients and family members on readmission rate	USA	Inpatients with end-stage kidney failure n=120	Single center RCT	**Intervention group** (n=60) Family consultation	**Control group** (n =60) Usual care	Family consultation occurred at patient's bedside<br>1) Physician reviewed patient and family understanding of events that caused the hospital admission<br>2) Assessed cognitive impairment<br>3) Discussed ways for the support person to assist the patient with his or her medication adherence<br>4) Tailored information about health and risk factors	**1EP:**<br>- Readmission (30d): 19/60 patients in the control group vs. 12/60 patients in the intervention group were readmitted within 30d, p=0.15<br><br>**2EP:**<br>- Readmission (180d): no difference between intervention and control group (39/60 in the control group vs. 38/60 in the intervention group), p=0.85<br>- A&E Reattendance (30d): 12/60 patients in the control group vs. 8/60 in the intervention group	good

© 2021 Becker C et al. *JAMA Network Open.*

Xiao, S et al	Omaha System-based discharge guidance improves knowledge and behavior in Mainland Chinese patients with angina who are not receiving interventional treatment: A randomized controlled trial	Japan Journal of Nursing Science	2018	Effectiveness of discharge guidance based on the theoretical framework of the Omaha System	China	Inpatients with Ischemic heart disease n=150	Single center RCT	**Intervention group** (n=75) Discharge guidance, based on the theoretical framework of the Omaha System	**Control group** (n=75) Usual care	Three days before discharge, nurses clarified patients' problems at discharge. According to this, they developed specific intervention strategies (e.g., teaching, counseling regarding illness and treatment, healthcare behavior). After implementation of the interventions, nurses assessed the problems every day until discharge and adjusted the intervention strategies according to any changes in the problems.	**1EP:**<br>- Knowledge (Diag, at discharge): mean knowledge score of 3.18 (0.89) in the control group vs. 4.10 (0.67) in the intervention group<br><br>**2EP:**<br>- Patients behavior mean score of 3.35 (0.85) in the control group vs. 4.48 (0.56) in the intervention group at discharge<br>- Patients scores in knowledge and behavior increased during hospitalization (3 timepoints)<br>- Increase in scores higher in intervention group than control group	poor
Barker, R	The Effects of a Video Intervention on Posthospitalization Pulmonary Rehabilitation Uptake. A Randomized Controlled Trial	Am J Respir Crit Care Med.	2020	To study the effect of a codesigned education video as an adjunct to usual care on posthospitalization rehabilitation uptake	UK	Inpatients with COPD n=198	Single center RCT	**Intervention group** (n=98) education video regarding pulmonary rehabilitation uptake as supplementary discharge information	**Control group** (n=98) Usual care	in addition to standard discharge procedure patients were asked to watch an a patients- codesigned education video; further patients had online access to watch the video after discharge	**1 EP:**<br>- rehabilitation uptake 90 days after discharge: no difference in uptake between control (41%) and intervention (34%) group, p=0.370<br><br>**2 EP:**<br>- quality of life 90 days after discharge: no difference between control and intervention group<br><br>- mortality 90 days after discharge: no difference between control group (2%) and intervention group, p=1.0<br><br>- readmission 90 days after discharge: no difference between control (15%) and intervention group (22%), p=0.871	good
Wilkin, Z et al	Effects of Video Discharge Instructions on Patient Understanding A Prospective, Randomized Trial	Advanced Emergency Nursing Journal	2020	To evaluate the effects of video discharge instructions, as an adjunct to standard discharge procedures, on adult ED patient understanding of their discharge instructions	USA	Emergency Patients with upper respiratory tract infection, pharyngitis or gastroenteritis n=60	Single center RCT	**Intervention group** (n=30) education video containing discharge information regarding their disease	**Control group** (n=30) Usual care	video contained structured information regarding patients' disease	**1EP:**<br>- Discharge Knowledge (Discharge knowledge score): Patients in the intervention group had significant higher knowledge scores than patients in the control group (4.533 vs. 4.0, p=0.009)	poor

© 2021 Becker C et al. *JAMA Network Open.*

Ebrahimi, Hossein	The role of peer support education model on the quality of life and self-care behaviors of patients with myocardial infarction	Patient Education and Counseling	2020	To assess effect of peer education on the quality of life and self-care behaviors of patients with myocardial infarction	Iran	Inpatients with myocardial infarction, n=70	Single center RCT	**Intervention group** (n=35)<br>two one-hour training sessions	**Control group** (n=35)<br>Usual care	2 patients with a history of myocardial infarction acting as peers and trained patients regarding (i.e. definition of myocardial infarction, mechanism and cause of the symptoms, risk factors, general principles of the treatment, drug therapy, non-pharmacological management, physical activity, marital relationship, weight control, diet regimen, follow-up care, management of dyspnea, fatigue, and chest pain	**1EP**:<br>Quality of life: patients in the intervention group had a significantly higher quality of life than patients in the control group (p<0.001)<br><br>**2EP:**<br>patients' self-care behavior: mean score of self-care behaviors in the experimental group was significantly higher compared to the control group (p=0.003)	good
Doyle, S et al	Effect of personalised, mobile-accessible discharge instructions for patients leaving the emergency department: A randomised controlled trial	Emergency Medicine Australasia	2020	TO ass	Australia	emergency patients with back and abdominal pain n=60	Single center RCT	**Intervention group** (n=30)<br>personalised printed and mobile-accessible discharge instructions	**Control group** (n=30)<br>Usual care	Patients in intervention group additionally received personalized printed and mobile accessible discharge instructions. Content was based on local and national pain relief advice material with accompanying pictograms. Information on common side effects was given for each medication with accompanying weblinks to further comprehensive consumer medication advice.	1 EP<br>- Pain score: no significant difference in pain scores between the control and intervention groups<br><br>2EP<br>- Satisfaction (5d): intervention group patients had significantly higher odds of being ‘very satisfied’ compared to the control group (OR 7.14, 95% CI 1.18–50.00, p=0.015)<br><br>- Unscheduled GP follow-up visits: no difference between intervention and control group (p=0.706)	poor
Interventions: Specific communication techniques (Shared decision-making, Motivational interviewing, teach-back)												

© 2021 Becker C et al. *JAMA Network Open.*

Hess, EP et al	The chest pain choice decision aid: a randomized trial	Circulation Cardiovascular quality and outcomes	2012	To test the effect of a decision aid on patient knowledge, patient engagement in decision making and proportion of patients admitted to hospital	USA	Emergency patients with nontraumatic chest pain n=208	Single center RCT	**Intervention group** (n=101)<br>Decision aid (pictograph with the pretest probability of an acute coronary syndrome), shared decision making	**Control group** (n=103)<br>Usual care	Decision aid describing the rationale and results of the initial evaluation (ECG, troponin), the rationale for further cardiac stress testing, depicting on a pictograph the patient's pretest probability for ACS within 45 days and indicating management options<br>Participating clinicians were oriented on how to use the decision aid prior to study.	**1EP:**<br>- Knowledge (Diag, immediate post discharge survey): 3.0 (95% CI: 2.7-3.2) questions (out of 7) in the control group vs. 3.6 (95% CI: 3.4-3.9) in the intervention group were answered correctly, MD=0.67 (95% CI: 0.34-1.0)<br>- Knowledge (Diag, immediate post discharge survey): 1 patient (1%) in the control group vs. 24 patients (25%) in the intervention group correctly assessed their 45-d risk of ACS, p<0,0001<br><br>**2EP:**<br>- Decisional Conflict Scale (30d): 43.3 (95% CI: 32.2-39.6) in the control group vs. 22.3 (95% CI: 18.1-26.4), MD=-13.6 (95% CI: .19.1 to -8.1)<br>- Trust in physician (30d): 79.3 (95% CI: 75.4-83.2) in the control group vs. 83.4 (95% CI: 79.4-87.3) in the intervention group, MD 4.1 (95% CI: -1.4 to 9.6)<br>- Patient involvement (OPTION-Scale): 7.0 (95% CI: 5.9-8.1) in the control group vs. 26.6 (95% CI: 24.9-28.2) in the intervention group, MD=19.6 (95% CI: 1.6-21.6)<br>- Patient satisfaction (30d): 40% of patients in the control group vs. 61% of patients in the intervention group were satisfied (95% CI: 7%-33%)<br>- A&E Reattendance (30d): 0 patients in the control group vs. 3 in the intervention group reattended an ED, p=0.1195<br>- Readmission (30d): 0 patients in the control group vs. 2 patients in the intervention group were readmitted to hospital, p=0.2439<br>- Admission to cardiac observation unit: 77% of patients in the control group vs. 58% in the intervention group decided to be admitted do the observation unit for stress testing, p<0.0001<br>- Adverse events (30d): 0 in the control group vs. 0 in the intervention group<br>- Death (30d): 0 in the control group vs. 0 in the intervention group	good

© 2021 Becker C et al. *JAMA Network Open.*

Griffey, RT et al	The impact of teach-back on comprehension of discharge instructions and satisfaction among emergency patients with limited health literacy: A randomized, controlled study	Journal of communication in healthcare	2015	Impact of teach-back on comprehension of discharge instructions and satisfaction of patients with LHL	USA	Emergency patients with low health literacy n=254	Single center RCT	**Intervention group** (n=127) Teach-back regarding discharge instructions	**Control group** (n =127) Standard discharge procedure	Patients with low health literacy level (REALM) were eligible for inclusion teach-back regarding discharge instructions	**1EP:** Knowledge of different dimensions (after discharge):<br>- No difference regarding knowledge of diagnosis (47.3% in the control group vs. 54.6% in the intervention group, p=0.2)<br>- More patients in the intervention group knew their post-ED medications compared to the control group, without reaching statistical significance (48.2% in the control group vs. 65.4% in the intervention group, p=0.054)<br>- Significant difference in comprehension of post-ED care, post-ED selfcare (p <0.02) and post-ED follow-up (p<0.0001)<br><br>**2EP:**<br>- Satisfaction with quality of instructions (at discharge): 95/110 in the control group vs. 89/107 in the intervention group were satisfied, p=0.85 (no significant difference between intervention and control group)	poor

© 2021 Becker C et al. *JAMA Network Open.*

Hess, E. et al	Shared decision making in patients with low risk chest pain: prospective randomized pragmatic trial	BMJ	2016	To test the effectiveness of the decision aid to improve patient knowledge and decrease unnecessary resource use	USA	Emergency patients with chest pain n=898	Multicenter RCT	**Intervention group** (n=451) Shared decision-making	**Control group** (n=447) Usual care	Use of a Cats plot as a design aid depicting risk of having a heart attack within the next 45 days	**1EP:**<br>- Knowledge (Diag, immediate post visit survey) in control group 3.6 (1.5) out of 8 questions vs. 4.2 (1.5) in the intervention group (OR 0.66; 95%CI 0.46 to 0.86) were answered correctly<br>- Knowledge (Diag, immediate post discharge survey): 2 patients (0.4%) in the control group vs. 10 patients (2.2%) in the intervention group correctly assessed their 45-d risk of ACS, p=0.039<br><br>**2EP:**<br>- Decisional conflict scale (30d): control 46.4 (14.8) vs. intervention 43.5 (15.3), OR -2.9(-4.8 to -0.90)<br>- Trust in physician (30d)control 87.7 (16.0) vs. intervention 89.5 (13.4), OR 1.7; 95%CI (-0.2 to 3.6)<br>- Patient involvement (OPTION-Scale): control 7.9 (5.4) vs. intervention 18.3 (9.4), OR 10.3; 95%CI (9.1 to 11.5)<br>- Readmission (30d): 19 (4.5) in control group vs. 20 (4.8) in intervention group, p=0.884<br>- A&E Reattendance (30d): 39 (9.3%) in the control group vs. 52 (12.5%) in the intervention group visited an ED, p=0.156<br>- Adverse events (30d): usual care 0 (0.0) vs. 1 (0.2) intervention, p=0.998<br>- Death (30d): usual care 0(0.0) vs 0 (0.0), p=1<br>- Patient satisfaction (30d): 192/447 patients in the intervention group vs. 221/451 patients in the intervention group were satisfied	good

© 2021 Becker C et al. *JAMA Network Open.*

Eyler, R et al	Motivational Interviewing to Increase Postdischarge Antibiotic Adherence in Older Adults with Pneumonia	The Consultant pharmacist: the journal of the American Society of Consultant Pharmacists	2016	Effects of motivational interview on drug adherence performed by pharmacists	USA	Medical inpatients with pneumonia n=30	Single center RCT	**Intervention group** (n=16) Motivational interviewing-enhanced discharge care	**Control group** (n =14) Standard discharge procedure	Motivational interviewing and counseling on their antibiotics by a pharmacist Assessment of readiness of discharge and confidence in adherence	**1EP:** **-** Adherence (7d): 9/14 vs. 14/16 in the intervention group were adherent, no significant difference in adherence to antibiotic treatment between intervention and control group, p=0.14 **2EP:** - General satisfaction (30d): Patients were very satisfied with intervention (mean 4.9 on a Likert Scale from 1-5) - Readmission (30d): 29% of the control group vs. 25% in the intervention group were readmitted, no difference between intervention and control group (4 vs 4, p=0.83)	poor
Naderloo, H et al	Effects of Motivational Interviewing on Treatment Adherence among Patients with Chronic Obstructive Pulmonary Disease: a Randomized Controlled Clinical Trial	Tanaffos	2018	To examine the effects of motivational interviewing on treatment adherence among patients with COPD	Iran	Inpatients with COPD (<65 years) n=60	Single center RCT	**Intervention group** (n=27) 5 one-to-one MI sessions	**Control group** (n=27) 2 training sessions on lifestyle, respiratory chest physiotherapy, and medication use	5 one-to-one, 15 to 45 minutes motivational interviewing (MI) sessions in addition to 2 training sessions on lifestyle, respiratory chest physiotherapy, and medication use	**1EP:** - Adherence (30d): Total adherence treatment score (total = 200, 0-6 point Likert scale) was 136.19 (19.8) in the control group vs. 160.26 (20.9) in the intervention group, p=0.000 **2EP:** - Adherence (60d): Total adherence treatment score (total = 200, 0-6 point Likert scale) was 136.26 (24) in the control group vs. 158.48 (27.6) in the intervention group, p=0.003 - Death (30d): 1/30 patients in the control group vs. 0/30 in the intervention group died	fair

© 2021 Becker C et al. *JAMA Network Open.*

eTable 2. Risk Assessment by Cochrane Risk of Bias Tool

AUTHOR / YEAR	STUDY NAME	RANDOM SEQUENCE GENERATION	ALLOCATION CONCEALMENT	SELECTIVE REPORTING	OTHER BIAS	BLINDING OF PARTICIPANTS AND PERSONNEL	BLINDING OF OUTCOME ASSESSMENT	INCOMPLETE OUTCOME DATA	Overall quality
**Waggoner, DM et al 1981**	**Physician influence on patient compliance: a clinical trial**	Usage of randomly ordered cards	Randomization in unsealed envelopes	Insufficient information to permit judgement	Insufficient information to permit judgement	No / incomplete blinding	No blinding	Imbalance in numbers between intervention and control group might induce bias to results	POOR
**Baker, D et al 1991**	**Evaluation of drug information for cardiology patients**	Method of randomization not described	Insufficient information to permit judgement	Insufficient information to permit judgement	Study seems to be free of other sources of bias	Insufficient information to permit judgement	Insufficient information to permit judgement	Missing outcome data balanced across groups	POOR
**Raynor, DK et al 1993**	**Effects of computer-generated reminder charts on patients' compliance with drug regimens**	Insufficient information to permit judgement	Insufficient information to permit judgement	Insufficient information to permit judgement	Insufficient information to permit judgement	Insufficient information to permit judgement	Insufficient information to permit judgement	Missing outcome data balanced across groups	POOR
**Ben Said, M et al 1994**	**A comparative study between a computer-aided education (ISIS) and habitual education techniques for hypertensive patients**	Insufficient information to permit judgement	Insufficient information to permit judgement	Insufficient information to permit judgement	Study seems to be free of other sources of bias	No blinding	No blinding	Missing outcome data balanced across groups	POOR
**Esposito, L et al 1995**	**The effects of medication education on adherence to medication regimens in an elderly population**	Shuffling cards or envelopes	Randomization was concealed with envelopes	Insufficient information to permit judgement	Study likely to be underpowered, only 20% of anticipated participants recruited	Insufficient information to permit judgement	Insufficient information to permit judgement	Insufficient information to permit judgement	POOR

© 2021 Becker C et al. *JAMA Network Open.*

**Smith, L et al 1997**	**An investigation of hospital generated pharmaceutical care when patients are discharged home from hospital**	Method of randomization not described	Insufficient information to permit judgement	Insufficient information to permit judgement	Study seems to be free of other sources of bias	Insufficient information to permit judgement	Blinding of outcome assessment ensured	Reasons for missing outcome data unlikely to be related to true outcome / Missing outcome data balanced across groups	**POOR**
**Hayes, KS et al 1998**	**Randomized trial of geragogy-based medication instruction in the emergency department**	Coin tossing	Coin tossing	Insufficient information to permit judgement	Study seems to be free of other sources of bias	Participants and personnel were blinded until the disposition had to be determined	No blinding	Reasons for missing outcome data unlikely to be related to true outcome / Missing outcome data balanced across groups	**POOR**
**Strobach, D et al 2000**	**Patient medication counseling-Patientenberatung zur Entlassungsmedikation**	List of random numbers	Open random allocation schedule (list of random numbers)	Insufficient information to permit judgement	Study seems to be free of other sources of bias	Insufficient information to permit judgement	Insufficient information to permit judgement	Data loss (18 lost to follow up) and Imbalance in numbers between intervention and control group might induce bias to results	**POOR**
**Davies, M et al 2001**	**Evaluation of a hospital diabetes specialist nursing service: a randomized controlled trial**	Method of randomization not described	Insufficient information to permit judgement	Insufficient information to permit judgement	Study seems to be free of other sources of bias	Insufficient information to permit judgement	Insufficient information to permit judgement	Loss of data, response rate of 47%, study underpowered	**POOR**
**Morice, AH. et al 2001**	**The role of the asthma nurse in treatment compliance and self-management following hospital admission**	Method of randomization not described	Insufficient information to permit judgement	Insufficient information to permit judgement	Study seems to be free of other sources of bias	Insufficient information to permit judgement	Insufficient information to permit judgement	Insufficient information to permit judgement	**POOR**

© 2021 Becker C et al. *JAMA Network Open.*

**Osman, LM et al 2002**	**A randomised trial of self-management planning for adult patients admitted to hospital with acute asthma**	Random numbers in sealed envelopes	Randomization was concealed with envelopes	Insufficient information to permit judgement	Study seems to be free of other sources of bias	Outcome measurement is not likely to be influenced by lack of blinding	Blinding of outcome assessment ensured	Reasons for missing outcome data unlikely to be related to true outcome / Missing outcome data balanced across groups	**GOOD**
**Gwadry-Sridhar, FH et al 2005**	**Pilot study to determine the impact of a multidisciplinary educational intervention in patients hospitalized with heart failure**	Randomization in blocks of four	Independent member of the research group did the randomizing	Insufficient information to permit judgement	Study seems to be free of other sources of bias	Outcome is not likely to be influenced by lack of blinding	Blinding of outcome assessment ensured	Reasons for missing outcome data unlikely to be related to true outcome / Missing outcome data balanced across groups	**GOOD**
**Koelling, TM et al 2005**	**Discharge education improves clinical outcomes in patients with chronic heart failure**	Usage of a computer random number generator	Treatment assignment was concealed from the patients and study personnel until after the randomization step.	Insufficient information to permit judgement	Study seems to be free of other sources of bias	Insufficient information to permit judgement	Outcome measurement is not likely to be influenced by lack of blinding (investigator aware of allocation, however outcome assessment was conducted in a scripted manner)	Reasons for missing outcome data unlikely to be related to true outcome / Missing outcome data balanced across groups	**FAIR**
**Manning, DM et al 2007**	**3D: a tool for medication discharge education**	Usage of a computer random number generator	Insufficient information to permit judgement	Insufficient information to permit judgement	Provided tool had to be completed individually at home by patients, intended effect could have been undermined Patients did not remember intervention	Outcome is not likely to be influenced by lack of blinding	Blinding of outcome assessment ensured	Loss of data, response rate <50%, imbalance in numbers between intervention and control (78 vs. 60)	**POOR**
**Cordasco, KM et al 2009**	**A low-literacy medication education tool for safety-net hospital patients**	Method of randomization not described	Insufficient information to permit judgement	Insufficient information to permit judgement	Selection and observation biases possible (poor follow-up)	Insufficient information to permit judgement	Outcome measurement is not likely to be influenced by lack of blinding	Data loss in both groups (follow-up 1 and 2) might induce bias to results	**POOR**

© 2021 Becker C et al. *JAMA Network Open.*

**Bladh, L et al 2011**	**Effects of a clinical pharmacist service on health-related quality of life and prescribing of drugs: a randomised controlled trial**	Usage of sequentially numbered, sealed envelopes	Randomization was concealed with envelopes and performed by two persons without knowledge about the study protocol	Insufficient information to permit judgement	Study seems to be free of other sources of bias	Insufficient information to permit judgement	Insufficient information to permit judgement	Data loss, only 53% of participants finished study	**POOR**
**Koonce, TY et al 2011**	**A pilot study to evaluate learning style-tailored information prescriptions for hypertensive emergency department patients**	Usage of a permuted block design with random block sizes of 2, 4, and 6 and consecutively numbered, sealed, opaque envelopes	Consecutively numbered, sealed, opaque envelopes	Insufficient information to permit judgement	Study seems to be free of other sources of bias	Outcome is not likely to be influenced by lack of blinding	Insufficient information to permit judgement	Missing outcome data balanced across groups	**FAIR**
**Sáez De La Fuente, J et al 2011**	**Efficiency of the information given at discharge and adherence of polymedicated patients**	Usage of a block randomization method	Allocation by block randomization method	Insufficient information to permit judgement	Study seems to be free of other sources of bias	Insufficient information to permit judgement	Blinding of outcome assessment ensured	Missing outcome data balanced across groups	**FAIR**
**Giuse, NB et al 2012**	**Using health literacy and learning style preferences to optimize the delivery of health information**	Usage of a block randomization method	Allocation by permuted block design with random block sizes of 2, 4, and 6	Insufficient information to permit judgement	Study seems to be free of other sources of bias	Insufficient information to permit judgement	Insufficient information to permit judgement	Missing outcome data balanced across groups	**POOR**
**Hess, EP et al 2012**	**The chest pain choice decision aid: a randomized trial**	Usage of a Web-based, computer-generated allocation sequence	Randomization was concealed with numbered envelopes	Study protocol available, pre-specified primary and secondary outcomes relevant to review reported (NCT01077037)	Study seems to be free of other sources of bias	Outcome is not likely to be influenced by lack of blinding	Blinding of outcome assessment ensured	Reasons for missing outcome data unlikely to be related to true outcome / Missing outcome data balanced across groups	**GOOD**

© 2021 Becker C et al. *JAMA Network Open.*

**Kommuri, NVA et al 2012**	**Relationship between improvements in heart failure patient disease specific knowledge and clinical events as part of a randomized controlled trial**	Usage of a random number generated by a computer program	Randomization was concealed	Insufficient information to permit judgement	Study seems to be free of other sources of bias	Insufficient information to permit judgement	Outcome measurement is not likely to be influenced by lack of blinding (investigator aware of allocation, however outcome assessment was conducted in a scripted manner)	Missing outcome data balanced across groups	**FAIR**
**Perera, K et al 2012**	**Medium of language in discharge summaries: Would the use of native language improve patients' knowledge of their illness and medications? Journal of Health Communication**	Using the drawing lots method	Usage of a folded piece of opaque paper from a container that had 130 such papers	Insufficient information to permit judgement	Study seems to be free of other sources of bias	Outcome is not likely to be influenced by lack of blinding	Blinding of outcome assessment ensured	Reasons for missing outcome data unlikely to be related to true outcome / Missing outcome data balanced across groups	**GOOD**
**Press, VG et al 2012**	**Teaching the use of respiratory inhalers to hospitalized patients with asthma or COPD: A randomized trial**	Random allocation sequence generated by a biostatistician	Study investigators and research assessors (RAs) were masked to the intervention.	Study protocol available, pre-specified primary and secondary outcomes relevant to review reported (NCT01456494)	Insufficient information to permit judgement (study might be underpowered)	Outcome is not likely to be influenced by lack of blinding (Study investigators and research assessors (RAs) were masked to the intervention, patients were unblinded.)	Outcome measurement is not likely to be influenced by lack of blinding	Missing outcome data balanced across groups	**good**
**Sanchez Ulayar, A et al 2012**	**Pharmaceutical intervention upon hospital discharge to strengthen understanding and adherence to pharmacological treatment**	Usage of sequentially numbered, sealed envelopes derived of a list of random numbers	Randomization was concealed with numbered envelopes	Insufficient information to permit judgement	Study seems to be free of other sources of bias	Insufficient information to permit judgement	Insufficient information to permit judgement	Missing outcome data balanced across groups	**POOR**
**Marušić, S et al 2013**	**The effect of pharmacotherapeutic counseling on readmissions and emergency department visits**	Usage of sequentially numbered, sealed envelopes	Randomization was concealed with envelopes	Insufficient information to permit judgement	Study seems to be free of other sources of bias	Outcome is not likely to be influenced by lack of blinding	Blinding of outcome assessment ensured	No missing outcome data	**GOOD**

© 2021 Becker C et al. *JAMA Network Open.*

**McCarthy, ML et al 2013**	**Does providing prescription information or services improve medication adherence among patients discharged from the emergency department? A randomized controlled trial**	Using a block randomization created by data coordination staff	Randomization was concealed with envelopes	Study protocol available, pre-specified primary and secondary outcomes relevant to review reported (NCT01174706)	Study seems to be free of other sources of bias	Outcome is not likely to be influenced by lack of blinding	Insufficient information to permit judgement (details of follow-up unclear)	Missing outcome data balanced across groups	GOOD
**Shah M et al 2013**	**Diabetes transitional care from inpatient to outpatient setting: Pharmacist discharge counseling**	Patients were randomized in a 1:1 ratio	Insufficient information to permit judgement	Insufficient information to permit judgement	Study seems to be free of other sources of bias	Insufficient information to permit judgement	Insufficient information to permit judgement	Data loss, only 52 patients could be contacted at 90d	POOR
**de Oliveira-Filho, AD et al 2014**	**Improving Post-Discharge medication adherence in patients with CVD: A pilot randomized trial**	Minimization (computer program)	Minimization (computer program)	Study protocol available, pre-specified primary and secondary outcomes relevant to review reported (RBR-26ydc3)	Study seems to be free of other sources of bias	Outcome is not likely to be influenced by lack of blinding (patients were blinded, study personnel however not)	Outcome measurement is not likely to be influenced by lack of blinding as researchers responsible for analyzing study data were blinded to the group, the pharmacists who performed data collection however were not.	Missing outcome data balanced across groups	GOOD
**Lin, R et al 2014**	**Effect of a patient-directed discharge letter on patient understanding of their hospitalisation**	Method of randomization not described	Insufficient information to permit judgement	Insufficient information to permit judgement	Study seems to be free of other sources of bias	Outcome is not likely to be influenced by lack of blinding	Blinding of outcome assessment ensured	Reasons for missing outcome data unlikely to be related to true outcome / Missing outcome data balanced across groups	POOR
**Moss, R et al 2014**	**A nurse-led randomised controlled trial of a structured educational programme for patients starting warfarin therapy**	Usage of a block randomisation method performed by an independent third person	Randomization was concealed with envelopes	Study protocol available, pre-specified primary and secondary outcomes relevant to review reported (ISRCTN08016736)	Baseline sociodemographic details were not collected, selection bias can therefore not be excluded No baseline knowledge test was conducted which might bias the outcomes, as knowledge its the primary outcome	Insufficient information to permit judgement	Insufficient information to permit judgement	Missing outcome data balanced across groups	POOR

© 2021 Becker C et al. *JAMA Network Open.*

**Fuenzalida, C et al 2015**	**Nurse-led educational intervention in patients with atrial fibrillation discharged from the emergency department reduces complications and shortterm admissions**	Usage of an electronically created list of random numbers	Insufficient information to permit judgement	Insufficient information to permit judgement	Study seems to be free of other sources of bias	Outcome is not likely to be influenced by lack of blinding	Blinding of outcome assessment ensured	Missing outcome data balanced across groups	**FAIR**
**Adamuz, J et al 2015**	**Impact of an Educational Program to Reduce Healthcare Resources in Community-Acquired Pneumonia: The EDUCAP Randomized Controlled Trial**	Usage of a computer-generated block randomization method	Randomization was concealed with envelopes	Study protocol available, pre-specified primary and secondary outcomes relevant to review reported (ISRCTN39531840)	Study seems to be free of other sources of bias	Outcome is not likely to be influenced by lack of blinding (study personnel not blinded, treating staff however was blinded)	Outcome is not likely to be influenced by lack of blinding	Reasons for missing outcome data unlikely to be related to true outcome / Missing outcome data balanced across groups	**GOOD**
**Basger, B et al 2015**	**Impact of an enhanced pharmacy discharge service on prescribing appropriateness criteria: a randomised controlled trial**	Method of randomization not described	Randomization was concealed with envelopes	Study protocol available, pre-specified primary and secondary outcomes relevant to review reported (ACTRN12611000995976)	Study seems to be free of other sources of bias	Outcome is not likely to be influenced by lack of blinding (only one pharmacist, however no intervention in control group)	Insufficient information to permit judgement	Missing outcome data balanced across groups	**FAIR**
**Griffey, RT et al 2015**	**The impact of teach-back on comprehension of discharge instructions and satisfaction among emergency patients with limited health literacy: A randomized, controlled study**	Randomization upon an odd or even last digit in their medical record number	Allocation could be foreseen by usage of last digit in the medical record number	Insufficient information to permit judgement	Study seems to be free of other sources of bias	Outcome is not likely to be influenced by lack of blinding	Outcome measurement is not likely to be influenced by lack of blinding	Missing outcome data balanced across groups	**POOR**
**Moore, SJ et al 2015**	**Impact of video technology on efficiency of pharmacist-provided anticoagulation counseling and patient comprehension**	Randomization by a variable permuted blocks randomization	Usage of a concealed-variable permuted blocks randomization scheme	Insufficient information to permit judgement	Study seems to be free of other sources of bias	Outcome is not likely to be influenced by lack of blinding	Insufficient information to permit judgement	Less than 50% of patients could be contacted for follow-up, therefore the study was underpowered	**POOR**

© 2021 Becker C et al. *JAMA Network Open.*

**Hess, E. et al 2016**	**Shared decision making in patients with low-risk chest pain: prospective randomized pragmatic trial**	Usage of an online password protected randomization algorithm	Allocation concealed by password protection (central web-based allocation)	Study protocol available, pre-specified primary and secondary outcomes relevant to review reported (NCT01969240)	Study seems to be free of other sources of bias	Outcome is not likely to be influenced by lack of blinding	Outcome measurement is not likely to be influenced by lack of blinding	Reasons for missing outcome data unlikely to be related to true outcome / Missing outcome data balanced across groups	**GOOD**
**Chan, H-Y et al 2016**	**Evaluation of a tablet-based instruction of breathing technique in patients with COPD**	Usage of Excel's RAND function to generate a set of six random numbers and reordering the group ABABAB by ranking numbers 12 times repeatedly	Randomization was concealed with envelopes	Selective reporting bias: Pre-specified primary endpoint was reported as secondary outcome (NCT01931267)	Study seems to be free of other sources of bias	Outcome is not likely to be influenced by lack of blinding (patients were blinded, study personnel however not)	Blinding of outcome assessment ensured	Missing outcome data balanced across groups	**POOR**
**Eyler, R et al 2016**	**Motivational Interviewing to Increase Postdischarge Antibiotic Adherence in Older Adults with Pneumonia**	Usage of a random-number generator	Insufficient information to permit judgement	Insufficient information to permit judgement	Study seems to be free of other sources of bias	Insufficient information to permit judgement	Insufficient information to permit judgement	Reasons for missing outcome data unlikely to be related to true outcome / Missing outcome data balanced across groups	**POOR**
**Hill, B et al 2016**	**Automated pictographic illustration of discharge instructions with Glyph: impact on patient recall and satisfaction**	Insufficient information to permit judgement	Insufficient information to permit judgement	Insufficient information to permit judgement	Study seems to be free of other sources of bias	Outcome is not likely to be influenced by lack of blinding	Blinding of outcome assessment ensured	Reasons for missing outcome data unlikely to be related to true outcome / Missing outcome data balanced across groups	**POOR**
**Kato, NP et al 2016**	**How effective is an in-hospital heart failure self-care program in a Japanese setting? Lessons from a randomized controlled pilot study**	Usage of stratified blocked randomization with regard to age and NYHA class	Randomization conducted by randomization services, study nurse was sent the treatment assignment	Study protocol available, pre-specified primary and secondary outcomes relevant to review reported (UMIN000001715)	Study seems to be free of other sources of bias	Outcome is not likely to be influenced by lack of blinding (patients were blinded, study personnel however not)	Outcome measurement is not likely to be influenced by lack of blinding (nurse who collected the data and the nurses who visited patients for the intervention were never the same)	Only 19/38 patients reached for follow-up	**FAIR**

© 2021 Becker C et al. *JAMA Network Open.*

**Olives, TD et al 2016**	**Seventy-two-hour antibiotic retrieval from the ED: a randomized controlled trial of discharge instructional modality**	Usage of a random-number generator	Randomization was concealed: the randomization code was maintained by the study coordinator and the primary authors were blinded	Study protocol available, pre-specified primary and secondary outcomes relevant to review reported (NCT01775969)	Study seems to be free of other sources of bias	Insufficient information to permit judgement	Outcome is not likely to be influenced by lack of blinding	Missing outcome data balanced across groups	**GOOD**
**Press, VG et al 2016**	**Effectiveness of Interventions to Teach Metered-Dose and Discus Inhaler Techniques. A Randomized Trial**	Usage of block randomization method	Insufficient information to permit judgement	Study protocol available, pre-specified primary and secondary outcomes relevant to review reported (NCT01426581)	Study seems to be free of other sources of bias	Outcome is not likely to be influenced by lack of blinding	Outcome measurement is not likely to be influenced by lack of blinding	Reasons for missing outcome data unlikely to be related to true outcome / Missing outcome data balanced across groups	**GOOD**
**Sanii, Y et al 2016**	**Role of pharmacist counseling in pharmacotherapy quality improvement**	Usage of block randomization method	Insufficient information to permit judgement	Insufficient information to permit judgement	Insufficient information to permit judgement	Insufficient information to permit judgement	Insufficient information to permit judgement	Missing outcome data balanced across groups	**POOR**
**Biscaglia, S et al 2017**	**A counseling program on nuisance bleeding improves quality of life in patients on dual antiplatelet therapy: A randomized controlled trial**	Usage of a computer-generated list	Randomization was concealed with envelopes by an independent study coordinator	Study protocol available, pre-specified primary and secondary outcomes relevant to review reported (NCT02554006)	Study seems to be free of other sources of bias	Outcome is not likely to be influenced by lack of blinding	Outcome measurement is not likely to be influenced by lack of blinding	Reasons for missing outcome data unlikely to be related to true outcome / Missing outcome data balanced across groups	**GOOD**
**Castelli, MR et al 2017**	**Effect of a Rivaroxaban Patient Assistance Kit (R-PAK) for Patients Discharged With Rivaroxaban: A Randomized Controlled Trial**	Usage of block randomization method	Randomization in blocks	Insufficient information to permit judgement	Intervention possibly confounded by separate prescriptions (Xarelto StarterKit)	Insufficient information to permit judgement	Insufficient information to permit judgement	Missing outcome data balanced across groups	**POOR**

© 2021 Becker C et al. *JAMA Network Open.*

**Fuenzalida, C et al 2017**	**Long-term benefits of education by emergency care nurses at discharge of patients with atrial fibrillation**	Usage of an electronically created list of random numbers	Insufficient information to permit judgement	Insufficient information to permit judgement	Study seems to be free of other sources of bias	Outcome is not likely to be influenced by lack of blinding (no blinding, but only intervention group received intervention)	Blinding of outcome assessment ensured	Missing outcome data balanced across groups	**FAIR**
**Chakravarthy, B et al 2017**	**Randomized pilot trial measuring knowledge acquisition of opioid education in emergency department patients using a novel media platform**	Usage of a random-number generator	Insufficient information to permit judgement	Insufficient information to permit judgement	No baseline knowledge test was conducted which might bias the outcomes, as knowledge is the primary outcome	Insufficient information to permit judgement	Insufficient information to permit judgement	Missing outcome data balanced across groups	**POOR**
**Al-Hashar, A et al 2018**	**Impact of medication reconciliation and review and counselling, on adverse drug events and healthcare resource use**	Usage of a computer-generated table (STATA)	Randomization was concealed with envelopes	Study protocol available, pre-specified primary and secondary outcomes relevant to review reported (NCT02805270)	Study seems to be free of other sources of bias	Outcome is not likely to be influenced by lack of blinding (All steps in each arm were carried out by the same pharmacist for all patients.)	Blinding of outcome assessment ensured	Reasons for missing outcome data unlikely to be related to true outcome / Missing outcome data balanced across groups	**GOOD**
**Athar, MW et al 2018**	**The Effect of a Personalized Approach to Patient Education on Heart Failure Self-Management**	Usage of an online randomizer	Randomization was concealed with envelopes	Study protocol available, pre-specified primary and secondary outcomes relevant to review reported (NCT03488979)	Study seems to be free of other sources of bias	Outcome is not likely to be influenced by lack of blinding (only study sonographer unblinded)	Outcome measurement is not likely to be influenced by lack of blinding (follow-up telephone calls following a script to minimize bias)	Missing outcome data balanced across groups	**GOOD**
**Breathett, K et al 2018**	**Pilot randomized controlled trial to reduce readmission for heart failure using novel tablet and nurse practitioner education**	Insufficient information to permit judgement	Insufficient information to permit judgement	Insufficient information to permit judgement	Study likely to be underpowered, only 25% of anticipated participants recruited	Insufficient information to permit judgement	Insufficient information to permit judgement	Missing outcome data balanced across groups	**POOR**

© 2021 Becker C et al. *JAMA Network Open.*

**Jasinski, MJ et al 2018**	**Family consultation to reduce early hospital readmissions among patients with end-Stage renal disease: A randomized clinical trial**	Patients were randomized in a 1:1 ratio in blocks of 6 to 8 using a computer program (randomization.org)	Randomization was concealed with envelopes	Study protocol available, pre-specified primary and secondary outcomes relevant to review reported (NCT02504021)	Study seems to be free of other sources of bias	Outcome is not likely to be influenced by lack of blinding	Blinding of outcome assessment ensured	No missing outcome data	**GOOD**
**Marušić, S et al 2018**	**Impact of pharmacotherapeutic education on medication adherence and adverse outcomes in patients with type 2 diabetes mellitus: A prospective, randomized study**	Usage of an electronically created list of random numbers	Open random allocation schedule (list of random numbers)	Study protocol available, pre-specified primary and secondary outcomes relevant to review reported (NCT03438162)	Study seems to be free of other sources of bias	Insufficient information to permit judgement	Blinding of outcome assessment ensured	No missing outcome data	**POOR**
**Naderloo, H et al 2018**	**Effects of Motivational Interviewing on Treatment Adherence among Patients with Chronic Obstructive Pulmonary Disease: A Randomized Controlled Clinical Trial**	Usage of a block randomization method	Allocation by block randomization method	Study protocol available, pre-specified primary and secondary outcomes relevant to review reported (IRCT201604128650N7)	Study seems to be free of other sources of bias	Insufficient information to permit judgement	Insufficient information to permit judgement	Missing outcome data balanced across groups	**FAIR**
**Graabaek, T et al 2019**	**Effect of a medicine's management model on medication-related readmissions in older patients admitted to a medical acute admission unit-A randomized controlled trial**	Usage of a block randomization method	Randomization was concealed with envelopes	Insufficient information to permit judgement	Study seems to be free of other sources of bias	Outcome is not likely to be influenced by lack of blinding (no blinding, but all interventions were performed by the same pharmacist)	Outcome measurement is not likely to be influenced by lack of blinding	Missing outcome data balanced across groups	**GOOD**
**Xiao, S et al 2018**	**Omaha System-based discharge guidance improves knowledge and behavior in Mainland Chinese patients with angina who are not receiving interventional treatment: A randomized controlled trial**	Usage of a computerized block randomization method	Allocation by block randomization method (blocks of two to four)	Insufficient information to permit judgement	Insufficient information to permit judgement	Insufficient information to permit judgement	Insufficient information to permit judgement	Missing outcome data balanced across groups	**POOR**

© 2021 Becker C et al. *JAMA Network Open.*

**Yin, D et al 2020**	**The effect of inpatient pharmaceutical care on nephrotic syndrome patients after discharge: a randomized controlled trial**	Usage of an online randomizer	Central allocation (web-based)	Insufficient information to permit judgement	Study seems to be free of other sources of bias	Insufficient information to permit judgement	Insufficient information to permit judgement	Missing outcome data balanced across groups	**POOR**
**Barker, R 2020**	**The Effects of a Video Intervention on Posthospitalization Pulmonary Rehabilitation Uptake. A Randomized Controlled Trial**	Usage of a computer-generated allocation sequence	Minimization (computer program)	Study protocol available , pre-specified primary and secondary outcomes relevant to review reported (ISRCTN13165073)	Study seems to be free of other sources of bias	Outcome is not likely to be influenced by lack of blinding (intervention group patients were asked to watch a video)	Blinding of outcome assessment ensured	Missing outcome data balanced across groups	**GOOD**
**Wilkin, Z et al 2020**	**Effects of Video Discharge Instructions on Patient Understanding A Prospective, Randomized Trial**	Usage of an online randomizer	Central allocation (web-based)	Insufficient information to permit judgement	Study seems to be free of other sources of bias	Insufficient information to permit judgement	No blinding	No missing outcome data	**POOR**
**Ebrahimi, H et al 2020**	**The role of peer support education model on the quality of life and self-care behaviors of patients with myocardial infarction**	Usage of block randomization method	Allocation by block randomization method	Study protocol available , pre-specified primary and secondary outcomes relevant to review reported (IRCT20180711040432N1)	Study seems to be free of other sources of bias	Outcome is not likely to be influenced by lack of blinding	Blinding of outcome assessment ensured	No missing outcome data	**GOOD**

© 2021 Becker C et al. *JAMA Network Open.*

**Doyle, S et al 2020**	**Effect of personalised, mobile-accessible discharge instructions for patients leaving the emergency department: A randomised controlled trial**	Usage of block randomization method	Allocation by block randomization method	Study protocol available, pre-specified primary and secondary outcomes relevant to review reported (ACTRN12618000667213)	Staff was not blinded to allocation which might have influenced discharge procedure; intervention group received significantly more pain medication at baseline	No blinding	Blinding of outcome assessment ensured	No missing outcome data	**POOR**

LOW RISK OF BIAS	**GOOD**
HIGH RISK OF BIAS	**POOR**
UNCLEAR RISK OF BIAS	**FAIR**

© 2021 Becker C et al. *JAMA Network Open.*

eFigure. Flow of Studies Through the Review Process

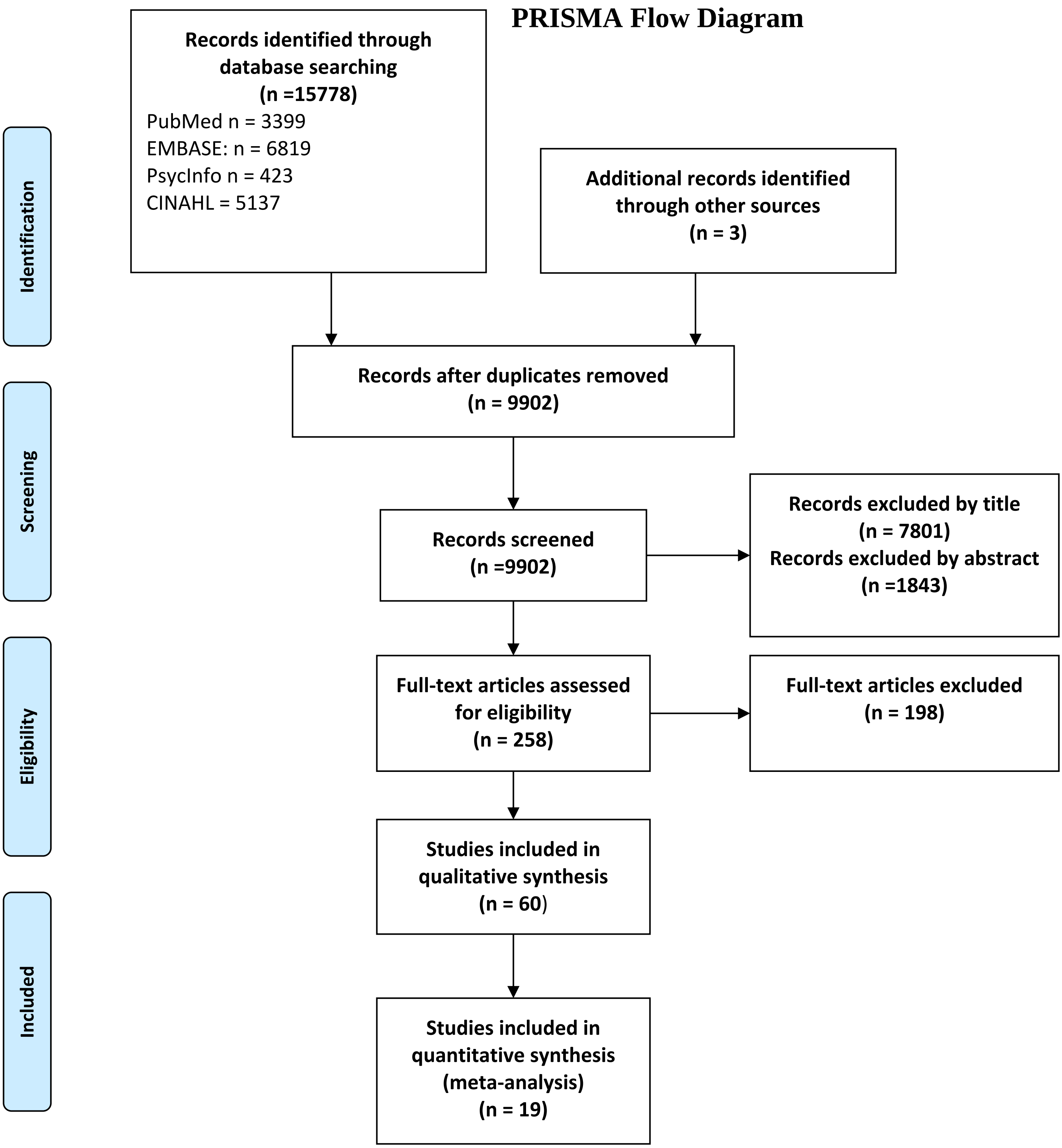

© 2021 Becker C et al. *JAMA Network Open.*
